# Molecular mechanism of mulberry response to drought stress revealed by complementary transcriptomic and iTRAQ analyses

**DOI:** 10.1186/s12870-021-03410-x

**Published:** 2022-01-17

**Authors:** Ruixue Li, Xueqiang Su, Rong Zhou, Yuping Zhang, Taichu Wang

**Affiliations:** Sericultural Research Institute, Anhui Academy of Agricultural Sciences, Hefei, Anhui China

**Keywords:** Mulberry, Drought stress, Transcriptomic, iTRAQ, Molecular mechanism

## Abstract

**Background:**

The use of mulberry leaves has long been limited to raising silkworms, but with the continuous improvement of mulberry (*Morus alba*) resource development and utilization, various mulberry leaf extension products have emerged. However, the fresh leaves of mulberry trees have a specific window of time for picking and are susceptible to adverse factors, such as drought stress. Therefore, exploring the molecular mechanism by which mulberry trees resist drought stress and clarifying the regulatory network of the mulberry drought response is the focus of the current work.

**Results:**

In this study, natural and drought-treated mulberry grafted seedlings were used for transcriptomic and proteomic analyses (CK vs. DS9), aiming to clarify the molecular mechanism of the mulberry drought stress response. Through transcriptome and proteome sequencing, we identified 9889 DEGs and 1893 DEPs enriched in stress-responsive GO functional categories, such as signal transducer activity, antioxidant activity, and transcription regulator activity. KEGG enrichment analysis showed that a large number of codifferentially expressed genes were enriched in flavonoid biosynthesis pathways, hormone signalling pathways, lignin metabolism and other pathways. Through subsequent cooperation analysis, we identified 818 codifferentially expressed genes in the CK vs. DS9 comparison group, including peroxidase (*POD*), superoxide dismutase (*SOD*), aldehyde dehydrogenase (*ALDHs*), glutathione s-transferase (*GST*) and other genes closely related to the stress response. In addition, we determined that the mulberry gene *MaWRKYIII8* (XP_010104968.1) underwent drought- and abscisic acid (ABA)-induced expression, indicating that it may play an important role in the mulberry response to drought stress.

**Conclusions:**

Our research shows that mulberry can activate proline and ABA biosynthesis pathways and produce a large amount of proline and ABA, which improves the drought resistance of mulberry. *MaWRKYIII8* was up-regulated and induced by drought and exogenous ABA, indicating that *MaWRKYIII8* may be involved in the mulberry response to drought stress. These studies will help us to analyse the molecular mechanism underlying mulberry drought tolerance and provide important gene information and a theoretical basis for improving mulberry drought tolerance through molecular breeding in the future.

**Supplementary Information:**

The online version contains supplementary material available at 10.1186/s12870-021-03410-x.

## Background

Humans have been producing silk products for at least 5000 years, and silk production is an ancient industry that is very popular in some Asian countries [1]. As the silkworm (*Bombyx mori*) is oligophagous, mulberry (*Morus alba*) leaves are its main food crop [2]. Mulberry belongs to the family Moraceae in terms of taxonomy, and it has more than 1000 cultivated varieties and a wide planting area in many Asian countries [3]. Through continuous research, people have found that mulberry leaves are rich in a variety of compounds that are beneficial to the human body (such as resveratrol, oxyresveratrol, and mulberroside A) [4, 5], which can promote the maintenance of the human matrix in a healthy state and help the human body actively cope with hypertension, hyperglycaemia, atherosclerosis and other diseases [1, 6]. Therefore, the use of mulberry leaves is not only limited to feeding silkworms but also occupies an important position in the field of food and health care.

As a common global climate disaster, drought causes 50% of the total yield reduction every year, and the direct economic loss is as high as 6-8 billion US dollars [7]. Drought will lead to plant leaf cell volume reduction, cell rupture and a lower ratio of palisade tissue to sponge tissue, resulting in irreversible damage [8, 9]. Fortunately, a drought stress response network composed of many genes is widely distributed in plants and enables plants to make a variety of changes to mitigate the effects of drought [8, 10]. For example, plants can respond to drought stress by regulating stomatal closure, and ABA is a hormone signal directly related to stomatal opening and closing [11]. Arabidopsis *CLE25* gene (*AtCLE25*) can be transported into leaves, stimulate the accumulation of ABA, cause stomatal closure, reduce water loss, and cope with drought [12]; *SmWD40-17*0 is an important drought-responsive gene in *Salvia miltiorrhiza*; it regulates the metabolism of ABA to mediate the opening and closing of stomata to affect the drought resistance of plants [13]. In addition, plants can improve their drought tolerance by regulating the synthesis of protective proteins, sugars, proline and antioxidants [14–17]. Genomics, transcriptomics and proteomics have become reliable and practical tools to explore the molecular mechanism of these plants in response to drought stress. Whole-genome analysis of the *Brassica napus* L. glutathione peroxidase (GPX) gene family showed that *BmGPX21* and *BmGPX23* were induced by abiotic stress and may be involved in the related stress response. The response proteins and metabolic pathways of drought tolerance in maize grains were studied by proteomic analysis, and important candidate genes were screened for subsequent functional validation [18].

At present, some studies on drought stress have been carried out in mulberry. For example, mulberry glutamate dehydrogenase (*GDH*) can improve the protein solubility and chlorophyll content in transgenic tobacco to increase the drought tolerance of transgenic plants [19]. A chloroplast drop-induced stress protein (*MaCDSP32*) can increase the drought tolerance of plants by regulating the activity of SOD and the concentration of proline and soluble sugar [20]. Overexpression of receptor for activated C kinase 1 (Rack1) protein in Arabidopsis decreased the drought tolerance of transgenic plants, which may play a role as a negative regulator of drought stress [21]. These studies mainly focused on the regulation of certain specific genes on plant drought tolerance, although they did not identify drought response transcripts at the whole-genome level, and revealed the interactions between different metabolic pathways that enhance plant drought tolerance. In rice, grape, maize and other species [22–24], transcriptome or proteome sequencing work under drought stress has been completed, and the strategies of these species to deal with drought stress have been revealed, which provides many gene resources for obtaining drought-tolerant varieties by molecular breeding. Therefore, it is necessary to study the characteristics of the mulberry response to drought and the molecular mechanisms of different metabolic pathways to improve drought tolerance via transcriptomics and proteomics.

In this study, mulberry leaves under normal growth and drought stress were used as the research objects, and the changes in related metabolites and key gene expression patterns of mulberry leaves under drought stress were analysed by transcriptome and proteome analyses. We identified 9889 differentially expressed genes (DEGs) and differentially expressed proteins 1893 (DEPs), of which 818 members showed codifferential expression. These codifferentially expressed genes were significantly enriched in drought stress-related metabolic pathways, such as antioxidant activity, hormone signal transduction pathway, and glutathione metabolism. In addition, through sequencing and qRT-PCR analysis, we identified many candidate genes responding to drought stress in mulberry. These results lay a foundation for elucidating the molecular mechanism of the mulberry response to drought stress.

## Materials and methods

### Plant materials

The mulberry materials used in this experiment were grown in Dayang Industrial Park (Hefei City, Anhui Province, China), which is a provincial mulberry germplasm resource nursery. The grafted mulberry seedlings in good growth conditions were maintained under the same water-fertilizer system and management plan during the cultivation period. When the mulberry tree entered the vigorous period, the leaf colour changed from light green to dark green, and the leaves were collected when they reached the maximum leaf area. At the same time, some mulberry trees were dehydrated to obtain mulberry leaves under drought stress. Normally growing mulberry seedlings and plants subjected to drought treatment for 0 day (d), 3 d, 6 d, 9 d, and 12 d were collected for physiological and biochemical index detection and RNA-seq and qRT-PCR experiments. In addition, grafted mulberry seedlings with good growth conditions were selected and sprayed with exogenous ABA on their leaves (both positive and negative leaves, ABA concentration of 0.02 mM). Then, leaf materials of 0 h, 2 h, 6 h, 8 h, 12 h, 24 h after treatment were collected for reserve. To eliminate experimental error, we set up three biological replicates for each leaf sample.

### Detection of relative water content and chlorophyll content in mulberry leaves

When measuring the relative water content of mulberry leaves, the leaves of normal growth and drought stress treatment (0 d, 3 d, 6 d, 9 d, and 12 d of drought treatment, each leaf was detected separately) were used as materials. Six parts of each mulberry leaf (0.5 g of mulberry leaves per share) were accurately weighed and quickly minced and used to determine the fresh weight of the leaves (W_f_). Then, three parts of mulberry leaves were placed in an oven at 120 °C for 1-2 h, and the dry weight was determined after drying (W_d_). The remaining three mulberry leaves were soaked in distilled water for 70 min, and the saturated fresh weight of leaves was recorded (W_T_). Finally, the relative water content of mulberry leaves was calculated by the following formula:

$$\mathrm{Relative}\;\mathrm{water}\;\mathrm{content}=\frac{{\mathrm W}_{\mathrm f}-{\mathrm W}_{\mathrm d}}{{\mathrm W}_{\mathrm T}-{\mathrm W}_{\mathrm d}}\times100\%$$

The maximum absorption peaks of chlorophyll a and b appears at 663 nm and 647 nm, respectively [25, 26]. In the determination of chlorophyll content in mulberry leaves, we also used normal growth and drought stress treatment (drought treatment 0 d, 3 d, 6 d, 9 d, 12 d) leaves as materials. For each plant material, 2 g of sample was used; 5 mL of acetone was added, and the sample was ground thoroughly. After grinding, 10 mL of acetone solution (80% acetone solution) was added to the sample and incubated for 3-5 min. Then, the solution after grinding was transferred to a centrifugal pipe (the volume was fixed to 15 mL) and centrifuged for 15 min at 3000 r/min. After centrifugation, the supernatant of the centrifuge tube was slowly aspirated, and acetone solution was used to fix the volume to 25 mL, which was the crude extract of mulberry leaf chlorophyll. After obtaining the crude extract of chlorophyll, the absorbance of the crude extract at 663 nm and 647 nm was measured by spectrophotometry to obtain the concentration of chlorophyll a (C_a_) and chlorophyll b (C_b_), as well as the total concentration of chlorophyll (C_T_) in leaves (the control group was 80% acetone solution). The total chlorophyll content in mulberry leaves can be calculated by the following formula:

$$\begin{array}{c}C_a=12.21A_{663}-2.81A_{645}\\C_b=20.13A_{645}-5.03A_{663}\\C_T=C_a+C_b\\W_T=\frac{C_T\left(mg/L\right)/1000\times V\left(ml\right)/1000}m\end{array}$$

### Detection of peroxidase and catalase activity

The activity of the POD was determined using the guaiacol method, and 1 g of leaf sample was weighed, minced and placed into a mortar. After adding phosphoric acid buffer (0.05 mol/L, pH 5.5), the sample was fully ground into a homogenate. After grinding, the homogenate was transferred into a centrifuge tube and centrifuged for 10 min (3000 rpm/min). The supernatant was transferred into a volumetric flask, and the volume was set to 25 mL. The enzyme activity detection system was as follows: 9 mL of phosphoric acid buffer (0.05 mol/L, pH 5.5), 1 mL of H_2_O_2_ (2%), 1 mL of guaiacol solution (0.05 mol/L), and 0.1 mL of crude enzyme solution. The crude enzyme solution was heated and boiled as the control group. After the enzyme solution was added, the samples were immediately incubated in a 34 °C water bath for 3 min. The solution was diluted twice, and the absorbance at 470 nm was detected by UV spectrophotometry. The value was recorded every 1 min 5 times [27]. The value of A_470_ changing by 0.01 per min was 1 POD activity unit (U).

We used an ultraviolet absorption method to detect CAT activity [28]. One gram of sample was weighed for testing, phosphoric acid buffer solution (0.05 mol/L, pH 7.8) precooled at 4 °C was added to grind the sample into homogenate, the sample was transferred into a centrifuge tube, the volume was fixed to 10 mL, and the sample was allowed to stand in the refrigerator at 4 °C for 10 min. Then, the sample was centrifuged at 3000 rpm/min for 15 min, and the supernatant was collected to obtain crude enzyme solution. Afterwards, 1.5 mL of phosphate buffer, 1 mL of distilled water, and 0.2 mL of crude enzyme solution were added to the centrifuge tube during measurement (crude enzyme was heated and boiled as a control). The centrifuge tube was preheated at 25 °C, and 0.3 mL of hydrogen peroxide (0.1 mol/L) was added. The absorbance at 240 nm was detected immediately, and readings were recorded every minute for a total of 5 times.

### RNA extraction and cDNA library construction

The plant material used in this experiment was mulberry leaves, and RNA extraction was performed using a Tiangen (Beijing, China) RNA extraction kit. Reverse transcription was performed using a PrimeScriptTM^RT^ reagent kit with gDNA Eraser (Takara, Kusatsu, Japan), and each reaction consisted of 1 μg of RNA. With the help of Beacon Designer 7 software, qRT–PCR primers for mulberry were designed. The qRT-PCR system was 20 μL, including 10 μL of SYBR solution (Takara, Kusatsu, Japan), 6.4 μL of sterile water, 2 μL of cDNA solution, 0.8 μL of upstream primer and 0.8 μL of downstream primer. The mulberry *ACTIN* gene was used as an internal reference, and the qRT–PCR procedure was based on the results of Cao et al. [29] Three sets of repeats were performed for each sample, and the relative expression level of genes was calculated by the 2^-△△Ct^ method [30].

### Transcriptome sequencing

In this study, we sequenced the transcriptome of mulberry leaves that were treated with normal growth (CK, control group sample) and drought stress for 9 days (DS9, sample of experimental group). To eliminate errors caused by differences between samples, three biological replicates were set for the control group and the experimental group (a total of 6 cDNA libraries were constructed for subsequent transcriptome sequencing). After all cDNA libraries were successfully constructed, the Illumina HiSeq™ 2500 sequencing platform was used for paired-end sequencing, and the quality of the data was first checked after the original data were obtained. We used Trimmomatic software (simple clip threshold = 12, minAdapterlength = 1) to filter the raw data [31] and remove adaptors, poly-n reads (more than 10% anonymous nuclei) and low-quality reads. Relatively accurate data and clean reads were obtained, and the contents of Q20, Q30 and GC in clean reads were calculated. Then, the mulberry genome was used as the reference genome, and the obtained clean reads were mapped to the reference genome (https://morus.swu.edu.cn/). Bowtie software (v2.2.3) was used to construct the reference genome index, and then with the help of TopHat (v2.0.12), the clean reads were aligned with the mulberry reference genome (mismatch = 2) [32]. Next, the FPKM value of unigenes (fragments per kilobases of exons per million mapped reads) obtained in the previous step was calculated by cufflinks software (V2.1.1) [33].

When analysing the differential expression of genes, two mulberry leaf samples were compared to identify the genes that were differentially expressed between the two samples. When identifying differentially expressed genes, the FPKM value method was used to analyse the transcriptional abundance of unigenes, which can effectively eliminate the influence of gene length and sequencing level on the unigene expression level. IDEG6 software was used to identify pairs of differentially expressed genes. The statistical results of multiple tests were all corrected using the Benjamini–Hochberg false discovery rate program (*P* value< 0.05). Finally, the formula log2 (fold change) was used to analyse unigenes in two samples to determine whether they were differentially expressed genes. Unigenes were upregulated when log_2_(fold change) > 1 and downregulated when log_2_(fold change) < − 1.

### Protein extraction from mulberry leaves

The extraction of mulberry leaf protein was carried out using the TCA/acetone method. First, the sample homogenate was centrifuged at 15000 rpm/min for 1 min (4 °C), and the precipitate was collected and freeze-dried in vacuo. The protein was extracted using the phenol buffer extraction method, and the supernatant portion of the mixture was collected. The obtained supernatant containing the protein extract was concentrated and stored in an ultralow temperature refrigerator for later use.

### Protein samples preparation and labelling

After obtaining the protein extract, we weighed 200 μg of sample to be tested (the control group and the experimental group were evaluated separately), added 5 μL of DL dithiothreitol solution (1 mol/L), and incubated the samples at 37 °C for 1 h. Then, another 20 μL of indole-3-acetic acid solution (1 mol/L) was added and incubated at 25 °C for 1 h in the dark. The precipitate was centrifuged, and 100 μL of urea solution was added and centrifuged again to collect the precipitate (this operation was repeated twice). Then, 100 μL of triethylamine borane solution was added to the precipitate, mixed and centrifuged to collect the precipitates (this operation was repeated three times). After that, trypsin was added to the precipitate (precipitate:enzyme solution = 50:1) and hydrolysed at 37 °C for 12 h.

After enzymolysis, the protein was labelled according to iTRAQ 8-plex reagent kits. After adding isopropanol to each vial of iTRAQ reagent, 113, 114, 115 and 116, 117, 118 iTRAQ reagents were used to treat CK and DS9 sample protein solutions (incubated at 25 °C for 2 h). After labelling, water was added to stop the reaction. Each centrifuge tube was vortexed to collect the solution, and iTRAQ analysis was performed after freeze-drying.

### Separation of peptides by LC–MS/MS

After collecting the enzymatically hydrolysed protein solution, we resuspended it in eluent. Peptide separation was performed in an Agilent 1200 HPLC (UV detection wavelengths: 210 nm and 280 nm), and the column was also purchased from Agilent (Wilmington, DE, USA). The specifications of the guard column and separation column are 4.6 × 12.5 mm 5-μm, narrow-bore 5 m 2.1 × 150 mm. When collecting eluent, only 8-52 min liquid was collected, and 4-5 tubes were combined for a total of 10 tubes of mulberry leaf solution (cryopreservation). The eluents were A water/ACN/FA (98:2:0.1, v/v/v) and B water/ACN/FA (5:95:0.1, v/v/v). Then, the analysis was performed on a fitted Triple TOF 5600 System (AB SCIEX). Samples were loaded on a capillary C18 trap column (3 cm × 100 μm) and then separated on an Eksigent NanoLC-1D plus system (AB SCIEX) by a C18 column (15 cm × 75 μm). The specific parameters were as follows: current speed, 300 nL/min; linear gradient, 70 min. When collecting information, the MS scanning time was 250 ms, and the production scans that exceeded a threshold of 150 counts per second (counts/s) with a 2-5 charge state were collected at most 35 times (each cycle = 2.5 s, dynamic exclusion = 18 s).

### Proteome sequencing analysis

The paragon algorithm in protein pilot software v.5.0 was used to analyse the mulberry leaf protein data. Sequencing results were mapped to the established mulberry protein database, and the peptide sequence was obtained [34]. The parameters of peptide≥2 and false discovery rate > 1% were used to screen credible protein sequences. Remove invalid values and reverse library data, and screen differentially expressed proteins based on reliable proteins. Differential protein screening (determined by the ratio in the treated samples and their corresponding untreated controls) was performed under the condition of fold changes > 1.2 or < 0.83 difference multiples and *p* < 0.05 threshold [35]. After the identification of differential proteins, GO enrichment analysis was carried out by using the Wego online website (http://wego.genomics.org.cn). The KEGG database was used to analyse the signal transduction and metabolic pathways involved in different proteins. Protein protein interaction analysis uses string protein database (https://cn.string-db.org/).

### Transcriptome and proteome sequencing cooperation analysis

The basic principle of proteome and transcriptome cooperation analysis is the central rule, and the transcriptome data of CK vs. DS9 were collected and sorted as a database. Differentially expressed proteins were identified in the database, the transcription levels were determined and compared with the expression levels of the corresponding proteins, and Pearson’s correlation coefficient was calculated for correlation analysis [36]. The main parameters were as follows: protein UniquePeptide, 1; protein fold changes > 1.2 or < 0.83; protein significant, Q-value < 0.05 (each repetition occurs at least once); log_2_(fold change) > 1 or < − 1; pathway significant [35].

When we annotated the functions of DEGs and DEPs, we also used the phyper function in RSEM software to enrich the functions [37]. In GO enrichment analysis, the *P* value of the GO term was obtained to correct the FDR. When the FDR value was less than 0.001, it was confirmed that the GO term was significantly enriched in DEGs or DEPs. In the pathway analysis, the enrichment factor value is the ratio of the number of annotated DEGs/DAPs in the pathway term to the number of annotated genes/proteins in the pathway term. The *P* value ranges from 0 to 1, and the smaller the *P* value is, the more significant the enrichment result is. In addition, the blast identity and blast evaluation were 100 and 1e-8, respectively.

## Results

### Phenotypic and physiological responses of mulberry leaves to drought stress

**Fig. 1:**
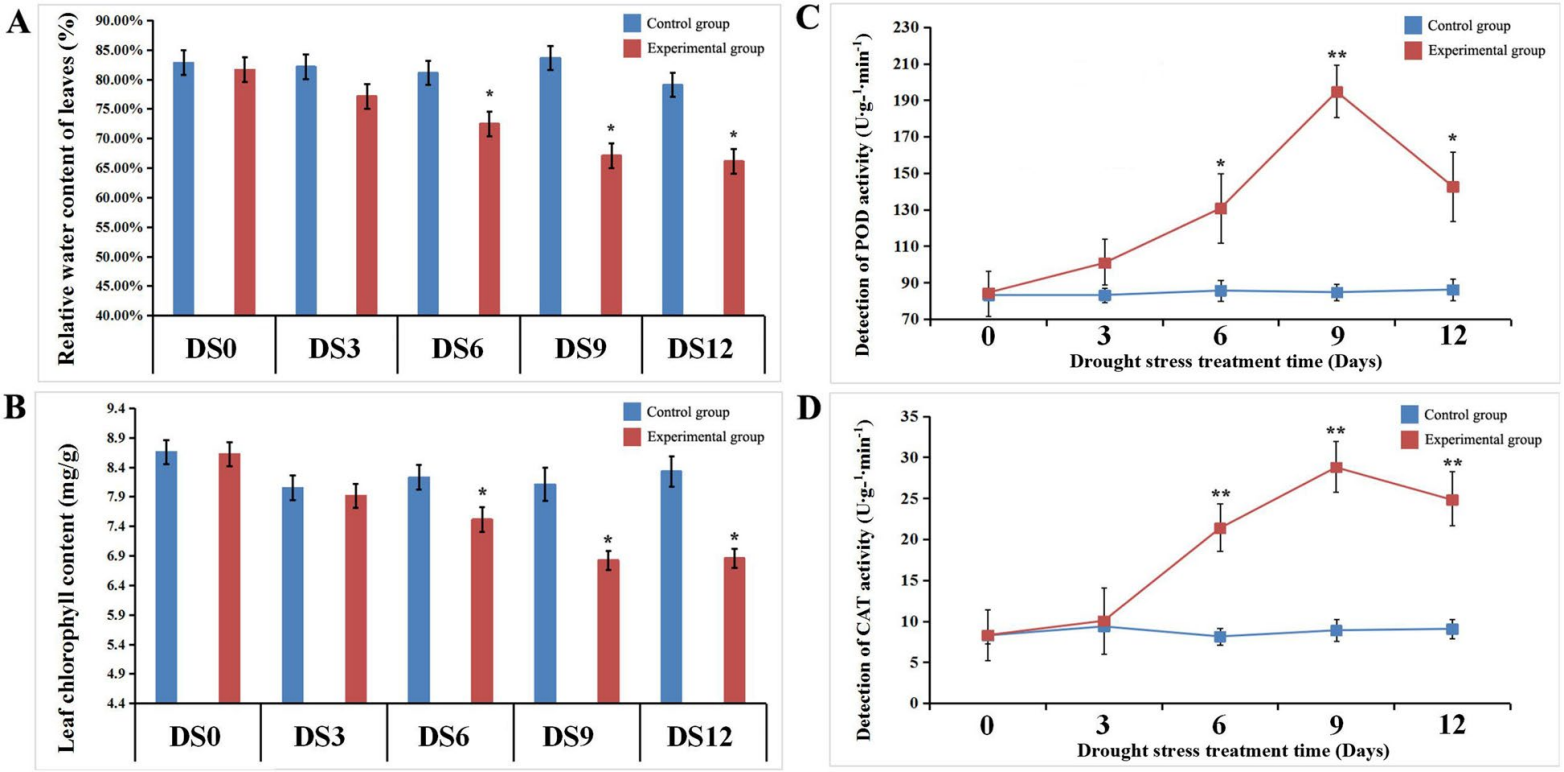
Analysis of physiological and biochemical indexes of mulberry leaves under drought stress. **a** Relative water content of mulberry leaves under drought stress. **b** Chlorophyll content of mulberry leaves under drought stress. **c** Analysis of POD activity in Mulberry Leaves under drought stress. **d** Analysis of CAT activity in Mulberry Leaves under drought stress. *, *P* ≤ 0.05; **, *P* ≤ 0.01

Drought stress can cause irreversible damage to all parts of plant tissues, leading to premature senescence, atrophy and insufficient nutrition supply, which disrupts the progression of photosynthesis and plant respiration. To explore the drought tolerance of mulberry, we first investigated the changes in physiological and biochemical indexes of mulberry leaves under the adverse environment of drought stress (Fig. 1). When mulberry was dehydrated, the relative water and chlorophyll contents of mulberry leaves decreased gradually. The leaf water and chlorophyll content decreased to 66.15% and 6.862 mg/g at 12 d, respectively, which was far lower than the level at 0 d (water content of 82.86% and chlorophyll content of 8.660 mg/g) (Fig. 1A, B). At the same time, the activities of peroxidase, catalase and other enzymes related to free radical scavenging increased gradually. The activities of POD and CAT reached a peak at 9 d of DS9, at 2.34 times (POD) and 3.47 times (CAT) higher than those of the control group (Fig. 1C, D). The results of physiological indexes showed that mulberry leaves began to exhibit some very obvious characteristics of the stress response after 9 d of drought treatment. Therefore, we selected mulberry leaves of this period (DS9) for transcriptome and proteome sequencing to analyse the molecular mechanism of mulberry drought tolerance.

### Quality analysis of transcriptome sequencing data

Through the investigation of morphological and physiological changes in mulberry leaves under drought stress, we found that the leaves of mulberry plants began to show obvious stress response characteristics after 9 d of drought treatment. At this time, the relative water content of leaves decreased significantly, and the activities of *SOD*, *CAT*, *POD* and other active oxygen free radical scavenging enzymes began to increase. Therefore, to further explore the molecular mechanism of the mulberry response to drought stress, we selected normal growth leaves (control group leaves, CK) and drought stress-treated leaves (leaves after 9 d of drought treatment, DS9) for transcriptome sequencing to analyse the regulatory network of mulberry leaf drought stress response (Table 1). For each sample, we set up three sets of biological repeats, so a total of 6 cDNA libraries were constructed (CK-1, − 2, − 3 and DS9-1, − 2, − 3). After sequencing on the Illumina 2500 sequencing platform, a total of 28.4 GB of original data were obtained and submitted to the NCBI short reading file (SRA) database with accession number PRJNA692033. According to the correlation analysis results of each sample, the three biological replicates of the same type of sample were highly correlated and could be used for subsequent analysis (Fig. S1).

**Table 1:** Overview of transcriptome sequencing data

ID	CK-1	CK-2	CK-3	DS9-1	DS9-2	DS9-3
Total Raw Reads	84,769,702	76,074,406	79,317,134	93,371,270	91,732,934	91,733,130
Total Clean Reads	76,331,066	68,472,362	71,999,802	84,869,778	84,157,852	84,442,344
Clean Reads Ratio	90.05%	90.01%	90.78%	90.90%	91.74%	92.05%
GC content	43.04%	43.30%	43.45%	42.47%	42.66%	42.84%
Q20	98.49%	98.47%	98.50%	98.58%	98.53%	98.52%
Q30	95.20%	95.15%	95.24%	95.42%	95.29%	95.27%

After processing the obtained raw data (low-quality and redundant sequences were deleted), a large number of clean reads were obtained, and the clean read ratio of 6 samples was between 90.01 and 92.05% (Table 1). The sample with the greatest number of clean reads was DS9-1, with 84,869,778 clean reads obtained. The CK-2 sample only produced 68,472,362 clean reads, which was the smallest component. In addition, the GC contents of the CK and DS9 samples were relatively similar, both approximately 43%. The Q20 and Q30 values of the CK and DS9 samples were also very close. The Q20 value of DS9-1 was the highest (98.58%), which was only 0.11% higher than that of CK-2, which had the smallest Q20. The Q30 values of the 6 samples (CK-1, − 2, − 3 and DS9-1, − 2, − 3) ranged from 95.15-95.42%. Then, we mapped the clean reads of CK and DS9 samples to the mulberry reference genome and obtained 26,223 and 25,963 genes, respectively. Among them, the CK and DS9 samples had 11,886 and 11,814 novel genes, respectively, which greatly enriched mulberry gene resources. The analysis of the FPKM value of gene expression showed that there was little change in the whole gene expression of mulberry leaves before and after drought treatment, and only the number of genes with FPKM values greater than 1 slightly increased after drought treatment (Fig. S2). Between the three biological replicates of CK and DS9 samples, gene expression levels were very similar (Fig. S3). Among the three samples of CK-1, − 2, and − 3, the number of genes with FPKM values greater than 10 accounted for 41.6% of the total, and approximately 21.4% of genes had FPKM values less than 1. In the three parallel biological repeat samples of DS9 (DS9-1, − 2, − 3), the number of genes with FPKM values less than 10 was similar, and the number of genes with FPKM values greater than 10 in the DS9-1 sample was less than that in DS9-2 and DS9-3 (Fig. S4). In general, the quality of transcriptome sequencing was high, and the correlation between three parallel biological repeats in each group was good, and the data could be used for subsequent in-depth analysis.

### Summary of proteome sequencing

A total of 878,525 spectra were identified in the CK and DS-9 samples by iTRAQ analysis, including 55,412 known spectra and 50,481 unique spectra (Table S1). Further analysis showed that 24,926 peptides and 23,442 unique peptides were found in our research, which constituted 6188 proteins (Table 2).

**Table 2:** Screening and identification of candidate genes involved in drought stress response in mulberry

Gene name	Genome ID	Drought stress response related genes		
		CK FPKM value	DS9 FPKM value	UP or DOWN
*MaLut5*	XM_010112737.1	88.34	36.76	DOWN
*MaZEP1*	XM_010090429.1	0	2.77	UP
*MaZEP2*	XM_010090427.1	0.88	0.145	DOWN
*MaZEP3*	XM_010090430.1	0.56	0.19	DOWN
*MaZEP4*	XM_010095339.1	38.84	70.08	UP
*MaNCED1*	XM_010094420.1	0.98	51.38	UP
*MaNCED2*	XM_010100822.1	0.06	0.5	UP
*MaNCED3*	XM_010109801.1	104.59	110.16	UP
*MaABA2-1*	XM_010108096.1	6.9	14.58	UP
*MaABA2-2*	XM_010101627.1	11.19	5.53	DOWN
*MaABA2-3*	XM_010114418.1	26.71	13.45	DOWN
*MaAOG1*	XM_010109589.1	8.08	17.48	UP
*MaAOG2*	XM_010112132.1	1.72	8.18	UP
*MaAOG3*	XM_010109603.1	6.81	19.27	UP
*MaAOG4*	XM_010109350.1	14.85	6.52	DOWN
*MaArg*	XM_010100630.1	19.75	50.11	UP
*MaP5CS1*	XM_010090276.1	4.71	54.15	UP
*MaP5CS2*	XM_010110091.1	0.67	3.49	UP
*MaproC*	XM_010108131.1	10.46	22.78	UP
*MaSOD1*	XM_010111635.1	171.54	35.22	DOWN
*MaSOD2-1*	XM_010110850.1	108.96	37.22	DOWN
*MaSOD2-2*	XM_010105331.1	59.75	31.51	DOWN
*MaPRX1*	XM_010107974.1	13.81	48.65	UP
*MaPRX2*	XM_010105049.1	131.88	270.39	UP
*MaPRX3*	XM_010106008.1	2.73	0.31	DOWN
*MaCAT1*	XM_010107694.1	15.18	20.91	UP
*MaCAT2*	XM_010096254.1	201.68	238.60	UP

The analysis of peptide length showed that the peptide with 7-13 amino acids was the largest, accounting for 63.55% of the total (Fig. S5a). In general, the length of the peptide started at 10 amino acids, and the proportion of peptide decreased with increasing amino acid quantity (Fig. S5b). By quantifying the lengths of 6188 protein sequences, we found that 20-30, 30-40, and 40-50 kD protein sequences accounted for the highest proportion, accounting for approximately 15% of the total protein. However, only 0.85% of the proteins were in the range of 0-10 kD, which was the smallest component. Most of the protein sequences identified by us were composed of 10 peptides, and only 383 proteins (6.19%) contained more than 11 peptides (Fig. S5c). In addition, the coverage rate of 4835 proteins was less than 20% (Fig. S5d).

### Screening and identification of differentially expressed DEGs and DEPs

After understanding the general situation for the transcriptome and proteome sequencing data, we screened DEGs and DEPs from these two types of sequencing data. DS9 and CK samples were compared and analysed, and DEGs were screened using the log_2_(fold change) formula: when log_2_(fold change) > 1, the unigene exhibits upregulated expression; and when log_2_(fold change) < − 1, the unigene exhibits downregulated expression. On this basis, we believe that log_2_(fold change) > 2 or log_2_(fold change) < − 2 are DEGs with very significant differences. The selection of DEPs was determined by the two conditions of fold change > 1.2 and q-value < 0.05. A total of 9889 DEGs were identified, including 6399 known genes and 3490 novel genes. In-depth analysis showed that the downregulated genes were the main component of these differentially expressed genes. We identified 6813 down-regulated genes (accounting for 68.9% of the total) and only 3076 up-regulated genes (Fig. S6).

To investigate the DEPs in mulberry leaves under drought stress, we screened the DEPs using proteome sequencing data. By pairwise comparison, 1893 DEPs were identified in CK vs. DS9 (Fig. S7). A total of 987 DEPs were upregulated, and 906 were down-regulated. Subcellular localization prediction showed that these DEPs were mainly located in the chloroplast, cytomatrix and nucleus, accounting for 81.3% of all DEPs. In contrast, 7 DEPs were enriched in the Golgi apparatus, nuclear envelope, and chloroplast membrane, only 0.37% of the total (Fig. S8).

### Functional enrichment analysis of DEGs and DEPs

**Fig. 2:**
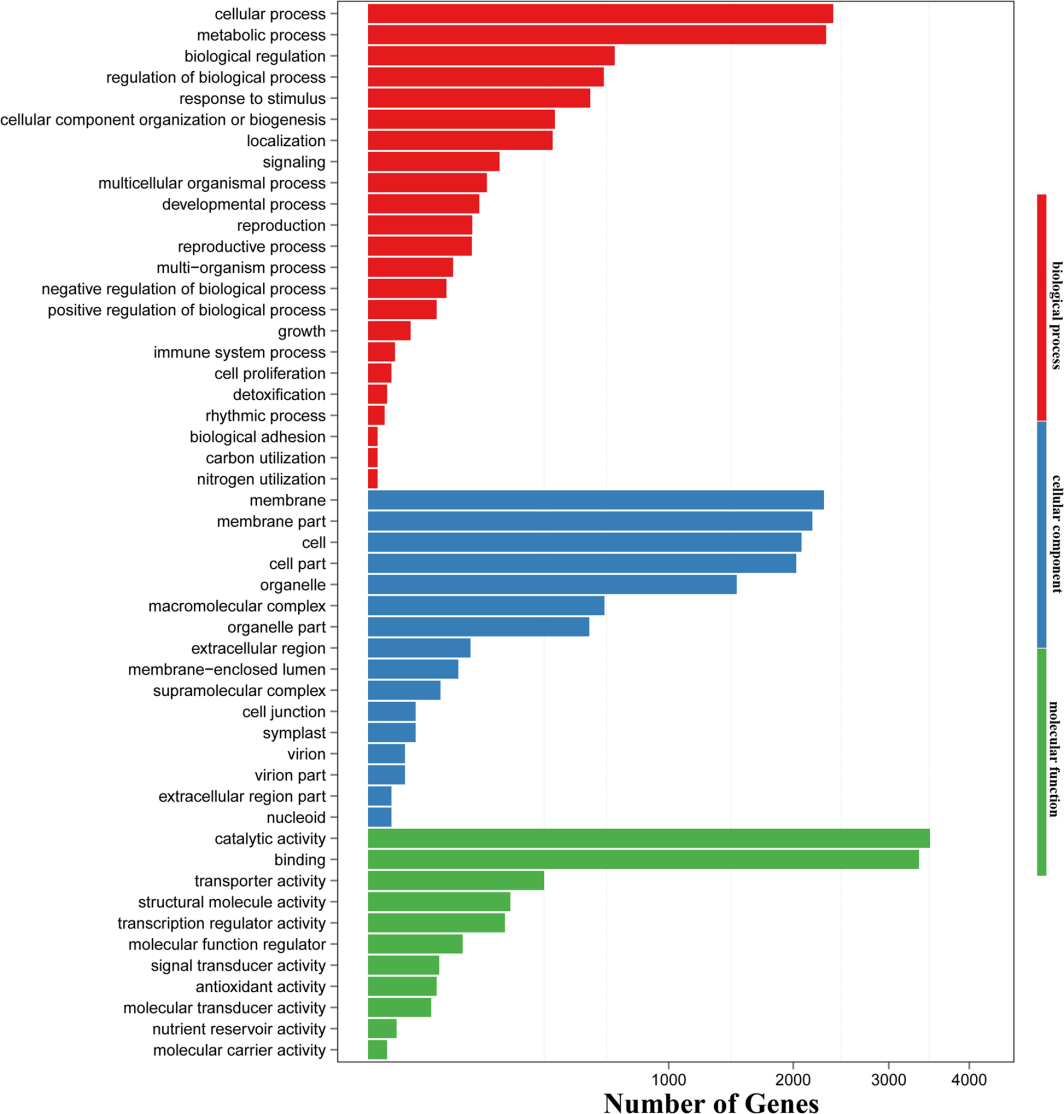
Go enrichment analysis of 9889 differentially expressed genes in CK vs. DS9 samples. Red is a biological process, blue is a cellular component, and green is a molecular function

After identifying the DEGs in the CK vs. DS9 comparison group, we attempted to analyse the functions of these DEGs. DEGs were searched in the GO database to determine the functional categories enriched by these genes. According to the GO enrichment results, the DEGs of the CK vs. DS9 comparison group were enriched in three main functional categories: biological process, cellular component, and molecular function (Fig. 2). The three main functional categories could be subdivided into 23, 16, and 11 small functional categories, respectively. Among biological processes, metabolic process and cellular process were the two main functional categories enriched. Membranes, membrane parts, cells, cell parts, and organelles were the functional categories of DEGs that were widely enriched in cellular components. The aggregation of DEGs in molecular function was relatively concentrated, mainly in catalytic activity and binding. In addition, we found that a large number of DEGs were enriched in biological regulation (GO:0065007), antioxidant activity (GO:0016209), transcription regulator activity (GO:0140110), and transporter activity (GO:0005215), which are closely related to the plant response to drought stress [38].

We used the KOG database to analyse DEPs in the CK vs. DS9 comparison group, and the results showed that these DEPs were mainly enriched in cellular processes and signalling, information storage and processing, and metadata. There were 353 DEPs that had no corresponding functional category, so they were classified into poorly characterized columns (Fig. S9). In addition, we found that DEPs were enriched in secondary metabolite biosynthesis, defence mechanisms, intracellular trafficking, and household signal transmission (XP_010086830.1, XP_010099626.1, XP_010105239.1, and XP_010106259.1, respectively). This finding indicates that there may be a large amount of secondary metabolite synthesis and hormone transduction in mulberry leaves when drought stress occurs, which can mobilize drought defence mechanisms to maintain life activities. Then, we investigated the functional classification of GO corresponding to these DEPs to understand the role of DEPs in the process of mulberry drought stress (Fig. S10). Similar to the transcriptome results, DEPs were mainly enriched in important functional classifications, such as metabolic process, binding, catalytic activity, cell part and cell. In the molecular function and biological process sections, we also found some functional categories related to drought stress response with abundant DEPs. For example, 259 DEPs were enriched in response to GO:0050896 (stimulus) and GO: 0065007 (biological regulation) in the biological process section. In the functional category of molecular function, DEPs in GO:0004871 (signal transducer activity), GO:0140110 (transcription regulator activity) and GO:0016209 (antioxidant activity) were mainly up-regulated, which indicated that the regulatory network responding to stress was activated and that reactive oxygen species (ROS) were eliminated in the leaves during drought.

### Correlation analysis between the proteome and transcriptome

**Fig. 3:**
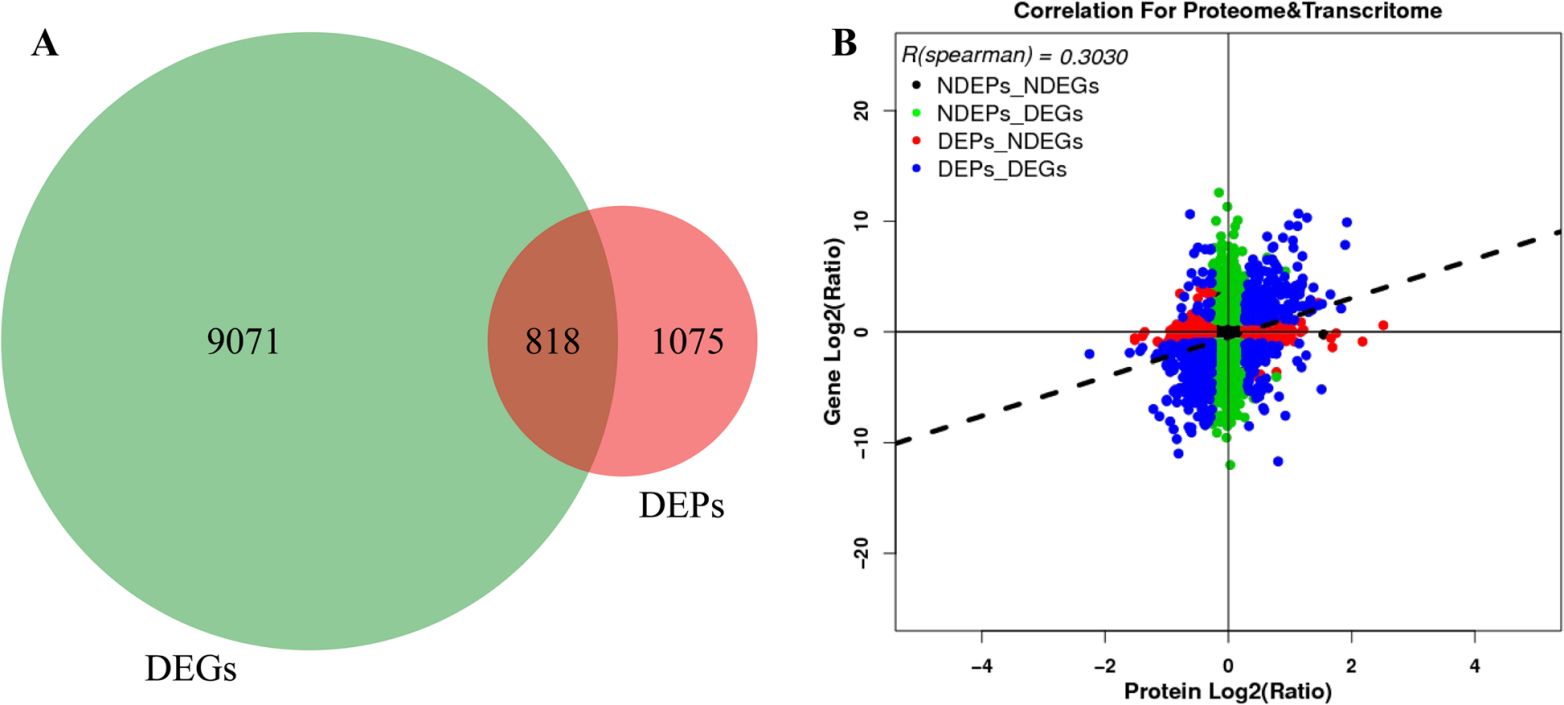
Correlation analysis of transcriptome and proteome. **a** Venn graph represents the number of association between DEGs and DEPs members. **b** All quantitative protein and gene expression correlation maps. The abscissa is the expression of protein expression level, and the ordinate is the transcriptional abundance of mRNA

To explore the relationship between differentially expressed proteins and their homologous genes, we performed correlation analysis of the sequencing data of CK and DS9 mulberry leaves (Fig. 3). A Venn diagram showed that only 818 members of 9889 DEGs and 1893 DEPs were associated with each other (Fig. 3a). Of the 818 codifferentially expressed members, 163 members showed the opposite trend at the transcription and translation levels (the transcript abundance decreased but the translation level was improved, or the reverse), and there were 28 upregulated DEGs and 135 downregulated DEGs. In addition, the transcription and translation of 655 co-differentially expressed genes showed the same trend (up or down at the same time) and were further subdivided into 430 down-regulated DEGs and 225 up-regulated DEGs. Then, we analysed the consistency between the transcription level and protein expression level of different groups by using the protein expression level as the abscissa and the gene expression level as the ordinate. The results showed that the correlation of the four components in NDEPs vs. NDEGs (no difference in transcription and translation), NDEPs vs. DEGs (no difference in protein content and significant difference in transcription level), DEPs vs. NDEGs (significant differences in protein content and no difference in transcription level), DEPs vs. DEGs (transcription and translation were significantly different) was poor, and the comprehensive Pearson correlation coefficient was only 0.303 (Fig. 3b).

Further analysis showed that there were 1192 genes and protein members in the NDEP vs. DEG group, which were mainly involved in pretranslational modification (Table S2). Examples included heat stress transcription factor (XP_010087101.1), E3 ubiquitin protein ligase (XP_010087488.1), and glutathione s-transferase U17 (XP_010096309.1). The DEP vs. NDEG group has 786 members, including serine/threonine-protein kinases, serine carboxypeptidase-like 48, ADP-ribosylation factor, GTPase-activating proteins and other genes involved in the plant response to abiotic stress (Table S3). Next, we analysed the GO functional categories in which DEGs and DEPs were enriched significantly at the same time (Fig. S11). The results show that carbohydrate metabolic process, glutathione metabolic process, oxidoreductase activity antioxidant activity, glutathione peroxidase activity and other functional categories are closely related to the response of plants to drought stress and are functional categories where DEGs and DEPs are simultaneously significantly enriched. In addition, a large number of codifferentially expressed genes, including hexokinase, UDP sugar pyrophosphorylase, cholestenol delta isomerase, and isoflavone 7-o-glucosyltransferase, were significantly enriched in phenoidal biosynthesis, steroid biosynthesis, isoflavonoid biosynthesis and other metabolic pathways and participated in the plant drought stress response (Fig. S12, Table S4).

### Identification of key genes for drought stress

Our research results show that as drought stress on the leaves gradually increases, some genes involved in the drought stress response gradually begin to upregulate their expression, such as proline biosynthesis and ROS scavenging-related genes. Plants in arid and unfavourable environments will produce a large amount of ROS, and the cells will be damaged. Therefore, we focused on genes related to free radical scavenging. The transcriptome and proteome were annotated to some free radical scavenging-related genes at the same time, including three *SODs*, three *PODs* and two *CATs* (Table 2). We found five genes encoding peroxidase, of which *MaPRX1, 2* showed an upregulated expression pattern in the CK vs. DS9 comparison group. We noticed that although *MaPRX2* is an upregulated gene, it also has a higher expression level in the control leaves. The expression level of *MaPRX1* in the CK was relatively low, while the expression level in the DS9 samples gradually increased (the FPKM value increased by 3.5 times). *MaCAT1*, *2* are two genes encoding CAT, both of which are up-regulated in the CK vs. DS9 comparison group. This finding indicates that there may be multiple genes encoding catalase in mulberry trees involved in the drought stress response. The three SOD genes we identified all showed low expression levels in DS9 samples. In the CK vs. DS9 comparison group, the proline biosynthetic pathway was also an up-regulated metabolic pathway, and *MaArg*, *MaP5CS1*, *MaP5CS2* and *MaproC* all showed up-regulated expression (Table 2).

In view of the role of the hormone signal transduction pathway in the response of plants to drought stress, we focused our attention on the mulberry hormone signal transduction pathway. Among various hormones, ABA is believed to play a major role in the response to drought stress (Table 2). In the ABA biosynthetic pathway of mulberry, we found 15 key enzyme genes that are differentially expressed in the transcriptome and proteome. These included *beta-ring hydroxylase* (*Lut5*), *zeaxanthin epoxidase* (*ZEP*), *9-*cis*-epoxycarotenoid dioxygenase* (*NCED*), *xanthoxin dehydrogenase* (*ABA*), and *abscisate beta-glucosyltransferase* (*AOG*). Except for *MaLut5*, we found members whose expression was upregulated in several other key enzyme genes. *MaZEP1*, *4* showed a significantly up-regulated expression pattern. The expression level of *MaNCED1* in the CK leaves was extremely low, while the transcription and translation levels in the mulberry leaves treated with drought stress increased significantly. *MaABA2-1* and *MaAOG1*, *2*, *3* also showed higher transcription and translation levels than CK in DS9 samples. These results show that after mulberry leaves are under drought stress, the ABA pathway is significantly activated, and the transcription and translation levels of some ABA biosynthesis-related enzyme genes are significantly increased. In the ABA signal transduction pathway, the transcription and translation levels of protein phosphatase 2C (*PP2C*) and ABA responsive element binding factor (*ABF*) increased (Table S5). These results show that the ABA transport pathway is activated, and ABA may begin to accumulate in mulberry leaves and open down-stream physiological responses, such as osmotic pressure regulation and stomatal closure control.

### qRT-PCR verification of key gene expression levels in mulberry under drought stress

After transcriptome and proteome sequencing data were used to screen mulberry drought stress-response genes, we analysed the expression patterns of these genes during the entire drought stress treatment, and the results showed that the qRT–PCR detection results were highly consistent with the sequencing results (Fig. S13). We found that the expression patterns of *MaSOD1* and *MaSOD2-2* were similar, and both had the highest expression level during the DS0 period. *MaSOD2-1* had a higher expression level in DS0-DS9, and the transcription peak appeared in the DS6 period. *MaPRX3* expression gradually decreased during drought stress, while *MaPRX1* and *2* were mainly expressed during DS0 and DS9. The difference is that *MaPRX1* also had a higher transcription level during DS3-DS6. Compared with other genes related to ROS removal, *MaCAT2* is mainly expressed in the late stage of drought stress treatment (DS6-12) and does not show transcriptional activity when the plant is initially exposed to adverse drought stress environments.

**Fig. 4:**
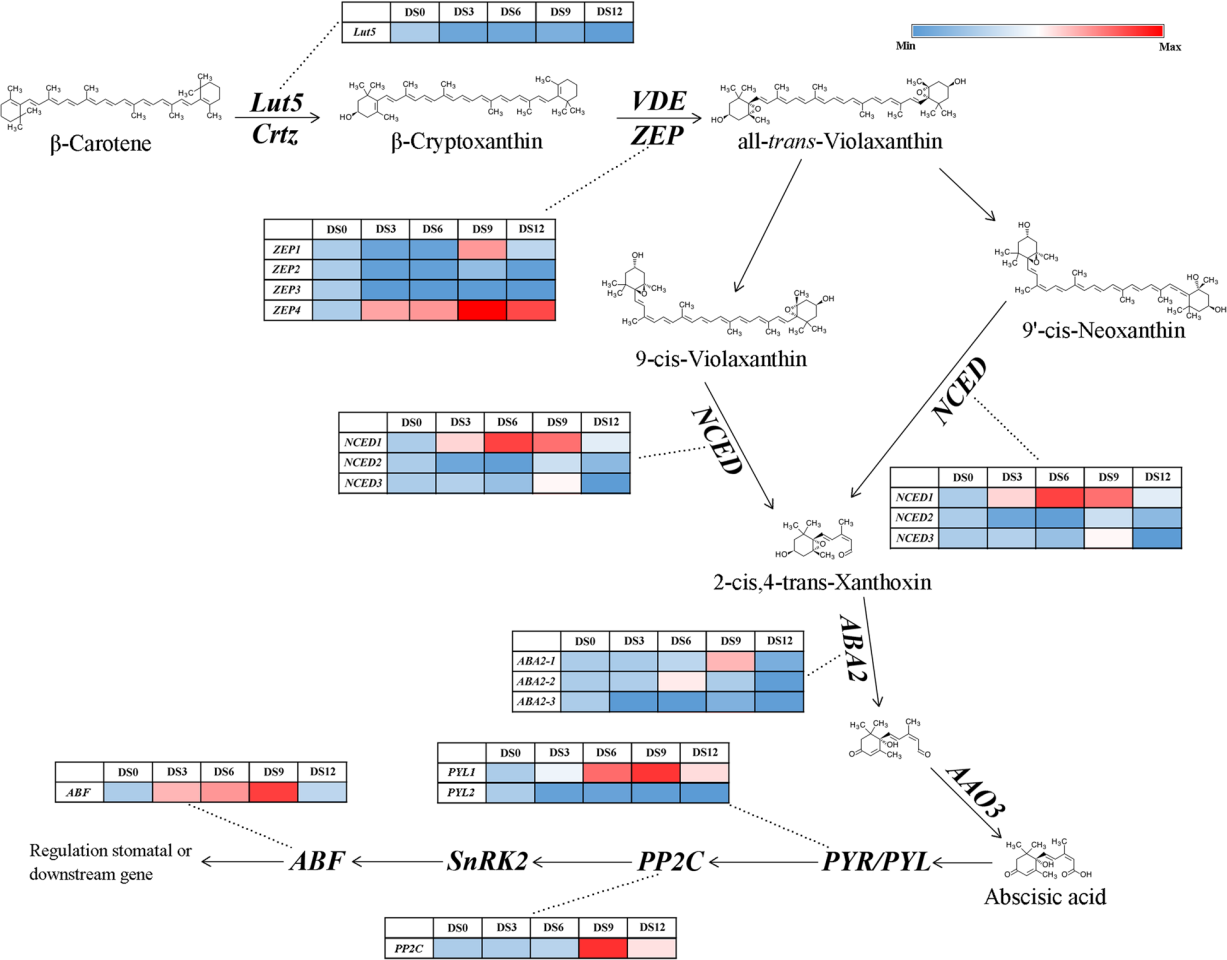
Heat map analysis of gene expression level of key enzymes in ABA biosynthesis

In the ABA biosynthesis pathway, most of the differentially expressed genes showed that at least one member was induced to expression by drought stress (Fig. 4). *MaLUT5* is an exception. We found that when mulberry leaves face drought stress, the expression of this gene is always at a low level, showing a pattern of down-regulation. Mulberry *MaZEP1* and *MaZEP4* were up-regulated in the CK vs. DS9 comparison group, but *MaZEP1* exhibited suddenly up-regulated expression at DS9, while *MaZEP4* exhibited gradually upregulated expression after the advent of drought stress and adverse environments and had an expression peak at DS9, followed by a decrease in expression. *MaNCED1* also showed a similar expression pattern, but the peak expression of *MaNCED1* was in the DS6 period (the expression level increased by 6.11 times compared with DS0), and then, the transcription level decreased significantly. *MaABA2-1*, *MaAOG1*, *2*, *3* were all highly expressed in DS9 (the expression level was 4.11-7.93 times higher than that of the CK), indicating that they may participate in the ABA biosynthesis process during this period.

Proline can not only participate in the osmotic adjustment of plant cytoplasm but also stabilize the structure of biological macromolecules, reduce cell acidity, regulate cell redox potential, and play an important role in the process of plants dealing with drought stress [39]. In the proline biosynthesis pathway, four key up-regulated genes, *MaArg*, *MaP5CS*1, *MaP5CS2*, and *MaproC* were identified (Table 2). These genes all showed high expression levels at DS9, and the FPKM values of *MaArg* and *MaP5CS1* reached 50.11 and 54.15, respectively. The qRT-PCR results confirmed the accuracy of the sequencing results, and the expression levels of *MaArg*, *MaP5CS1*, and *MaproC* increased with increasing drought stress treatment time, indicating that the expression of these genes was induced by drought stress (Fig. S14).

**Fig. 5:**
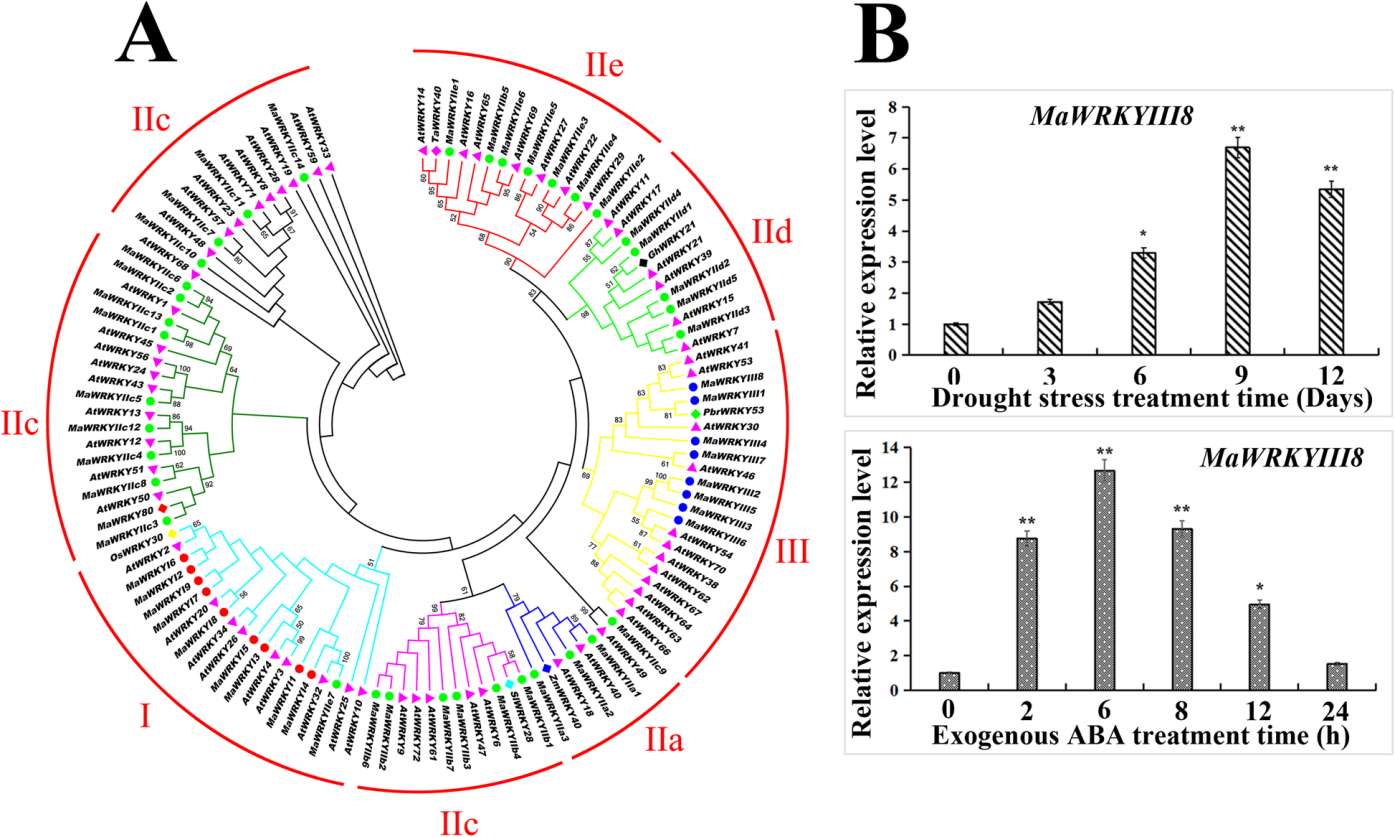
Phylogenetic relationships and subfamily designations in *WRKY* proteins from *Morus alba*, *Arabidopsis thaliana*. And analysis of *MaWRKYIII8* expression pattern. **a** Three types of *WRKY* genes are represented by GroupI-III in the figure. **b** Expression pattern of *MaWRKYIII8* induced by drought stress and exogenous ABA, the sampling time was 0d, 3d, 6d, 9d, 12d and 0 h, 2 h, 6 h, 8 h, 12 h, 24 h, respectively

In addition, we carried out genome-wide identification and compound evolutionary tree analysis of mulberry *WRKY* gene family, and found that *MaWRKYIII8* (XP_010104968.1) is closely related to *PbrWRKY53* and *AtWRKY57* (involved in plant drought stress response), it is speculated that *MaWRKYIII8* may also have similar biological functions (Fig. 5a, Table S6) [40, 41]. Interestingly, *MaWRKYIII8* was a co-upregulated gene in our sequencing analysis. String protein database analysis showed that it may be related to the regulation of E3 ubiquitin protein ligase and *MaWRKYIIc14* (Fig. S15). qRT-PCR results showed that the expression level of *MaWRKYIII8* gradually increased under drought stress (0 d, 3 d, 6 d, 9 d, 12 d), indicating that *MaWRKYIII8* expression was induced by drought (Fig. 5b). *MaWRKYIII8* expression was also activated by exogenous ABA treatment, reaching a peak at 8 h and then gradually decreasing (Fig. 5b). These results suggest that *MaWRKYIII8* expression is induced by drought and ABA, which may play an important role in the response of mulberry leaves to drought stress.

## Discussion

In the process of agricultural production, abiotic stresses such as drought, salinity, and high temperature are the main unfavourable environmental factors that affect the normal growth of crops and cause severe yield reduction. Among the various abiotic stresses of plants, drought is the most important stress factor. Statistics from the United Nations from 2005 to 2015 show that droughts worldwide have caused direct economic losses of 29 billion U.S. dollars in agricultural production in developing countries, ranking first among all abiotic stresses [7]. When plants are under drought stress, their normal growth is severely affected, mainly in terms of slow leaf growth, short plant height and weak root system vigour. Drought stress can cause problems such as inhibited seed germination, reduced cell volume, reduced water content, decreased chlorophyll content, premature ageing, or small and dense stomata [42, 43]. When mulberry trees were subjected to drought stress (9 d of drought treatment), the leaf water content decreased from 82.86 to 66.15%, and the chlorophyll content decreased from 8.660 mg/g to 6.862 mg/g (Fig. 1). At the same time, the accumulation of organic matter in leaves may be significantly affected [44]. Leaf is the main agricultural output of mulberry and can be used to feed silkworms or continue intensive processing. Premature leaf senescence caused by drought will cause serious loss of nutrients in mulberry leaves and loss of their usefulness. Therefore, reducing the impact of drought on the growth of mulberry trees and improving the ability of mulberry trees to resist drought stress are key issues that urgently need to be resolved in the sericulture industry.

This study used normal growth and drought stress (DS9) mulberry leaves as materials and conducted correlation analysis of the transcriptome and proteome in an attempt to discover the key genes that regulate the response of mulberry to drought stress. After correlation analysis, we found no strong correlation between proteins and transcripts (Fig. 3a, b). We identified 6188 protein sequences, of which 5117 (82.7% of protein sequence reads) were found in the transcriptome results. Although there are many sequences that can correspond between transcriptome and proteome data, this result is still lower than the results of studies in rice, tea plants and other species [25, 45]. However, the corresponding transcripts in transcriptome sequencing were not found for 1071 protein sequences, which may be because the half-life and stability of mRNA in mulberry changed in the drought stress environment. The 20,411 unigenes were only found in transcriptome sequencing, and no associated proteins were identified in the proteome. These results indicate that there may be a large number of transcriptional regulatory members among these genes (Fig. S16). In-depth analysis of DEGs and DEPs found that there were 818 member mRNAs and proteins in 9889 DEGs and 1893 DEPs that could be associated with each other (Fig. 3). The comprehensive Pearson correlation coefficient of NDEPs vs. NDEGs, NDEPs vs. DEGs, DEPs vs. NDEGs, and DEPs vs. DEGs was only 0.303, which indicated that there was only moderate consistency between the transcriptome and proteome, consistent with the results of moderate relevance between the mRNA and protein levels [46].

### The *SGR* gene is a potential enhancer of drought resistance in mulberry

According to previous studies and our results, plant leaves will lose their ability to stay green in a dehydrated environment [47]. In fact, some genes involved in the control of plant leaf greenness have been discovered, and their molecular mechanisms are relatively clear, such as stay green rice (*SGR*) genes and their homologous genes (*SGR1*, *SGR2*, and *SGR-like*) in *Arabidopsis thaliana* [48]. Previous studies have shown that *SGR1* and *SGR-like* can promote the degradation of chloroplasts, making the leaves appear yellow at an early time point [49, 50]. In contrast, SGR2 negatively regulates chloroplast degradation during abiotic stress and leaf senescence [51, 52]. We found homologous genes of *AtSGR1*, *AtSGR2*, and *AtSGR-like* in mulberry trees, and the results showed that XM_010101265.1 (*AtSGR1*) and XM_010107031.1 (*AtSGR-like*) were down-regulated, while XM_010109758.1 (*AtSGR2*) was up-regulated (Table S7). This finding indicated that mulberry leaves downregulated the SGR gene involved in chloroplast degradation in response to premature senescence and yellowing of mulberry leaves under drought stress. However, due to the antagonistic effect, XM_010109758.1 expression was significantly upregulated. This response improved the ability of mulberry to cope with premature leaf senescence, decreased the photosynthetic rate, maintained the normal morphology of chloroplasts, and enhanced its ability to respond to drought stress [53].

### Screening of genes related to stomatal regulation

When plants are in an unfavourable environment, the cell osmotic balance is broken, and proline is the more effective affinity osmotic adjustment substance for a certain class of plants [54]. Plants will increase the accumulation of proline in the body in response to drought stress to ensure the osmotic adjustment and balance of tissue cells [55]. Multiple genes related to the plant drought stress response can promote the accumulation of proline in transgenic plants to enhance the drought tolerance of plants. These include *CmLEA-S*, *PeSNAC-1*, and *MfbHLH38* [54, 56, 57]. In our study, both DEGs and DEPs were enriched in the proline biosynthetic pathway. The *MaArg*, *MaP5CS1*, and *MaproC* genes were up-regulated in the CK vs. DS9 comparison group (Table 2, Fig. S14). Among them, the transcription levels of *MaArg*, *MaP5CS1*, and *MaproC* increased with increasing drought stress treatment time and reached peak expression at DS9 (Fig. S14). The results of the upregulation of key genes for proline biosynthesis in mulberry trees under adverse drought stress environments are in line with tobacco and peach [58, 59]. The results in rootstocks subjected to drought stress were similar, indicating that these genes synthesize free proline to maintain the balance of cell osmotic pressure in response to drought stress in mulberry leaves. The stomata are openings in the aboveground part of plants, composed of two special guard cells, and are the key to plant respiration and photosynthesis. However, one of the ways for plants to improve drought tolerance is by regulating the opening and closing of stomata and their number and distribution to cope with adverse drought environments [60]. The opening and closing of the stomata is regulated by fine molecules, and under water shortage conditions, ABA is the main hormone signal factor that triggers the opening and closing of the stomata [61]. Our study showed that the expression of *MaZEP*, *MaNCED* and other ABA biosynthesis enzyme genes were significantly activated in mulberry DS9 samples (Fig. 4). At the same time, the expression levels of *PYL*, *PP2C* and *ABF* increased with increasing drought stress time (Fig. 4). Studies in rice showed that the overexpression of *PYL5* could enhance the drought tolerance of transgenic plants, but the normal growth of plants was inhibited [62]. Therefore, how to balance the relationship between stomatal regulation and plant growth requires further study.

### *MaWRKYIII8* may be involved in the response of mulberry to drought stress

A large number of studies have shown that transcription factors such as *MYB*, *bZIP*, and *WRKY* are involved in the response process of plant drought stress [63]. Among them, we found that *MaWRKYIII8* may have the potential function of participating in mulberry drought stress (Fig. 5). In recent years, many studies have investigated *WRKY* and its regulation of the drought resistance of plants. *HbWRKY82* (*Hevea brasiliensis*) is induced by exogenous ET (Ethrel) and ABA [64]. Heterologous overexpression of *HbWRKY82* in Arabidopsis can activate reactive oxidative species (ROS) clearance genes (*RbohD*, *CSD1*, *CSD2*, etc.) and hormone signalling genes (*EIN3*, *ABF3*, *ABF4*, etc.) and improve the dehydration tolerance of transgenic plants [60]. The strategy adopted by rice *OsWRKY30* to improve the drought tolerance of plants is to interact with genes in MAPK cascade signalling pathways, such as *OsMPK*3, *OsMPK14*, *OsMPK7*, and *OsMPK20-5,* and be phosphorylated by them, thereby regulating the drought tolerance of plants [65]. More importantly, *WRKY* transcription factors can also directly regulate the biosynthesis of ABA, thereby affecting the ability of plants to respond to stress. For example, *AtWRKY57*, *PbrWRKY53*, and *MaWRKY80* are all induced by drought and ABA, and by directly regulating the expression of downstream *RD29A*, *NCED*, *ABA3* and other genes, they affect the biosynthesis of ABA and mediate the response of plants to drought stress [21, 40, 66]. Therefore, we used natural drought and exogenous ABA to treat mulberry trees to examine the expression pattern of *MaWRKYIII8* (Fig. 5b). The qRT-PCR results showed that the expression level of *MaWRKYIII8* gradually increased under drought stress (natural dehydration for 0 d, 3 d, 6 d, 9 d, 12 d), indicating *MaWRKYIII*8 expression was induced by drought. When exogenous ABA was used to treat mulberry trees, *MaWRKYIII8* expression was also activated, reaching peak expression at 8 h, and then the expression level gradually declined (Fig. 5b). These results indicate that *MaWRKYIII8* expression is induced by drought and ABA and may play an important role in the response of mulberry leaves to drought stress. The function of *WRKY* transcription factors often depends on the W-box on the downstream gene promoter sequence [67]. Interestingly, when we examined the promoter sequences of key genes in the ABA biosynthetic pathway, we found several ABA-responsive cis-acting elements and W-box clusters on the promoter sequence of *MaNCED1* (Fig. S17), indicating that it may be a potential downstream regulatory gene of *MaWRKYIII8*.

We examined the sequence of the *MaWRKYIII8* promoter and found that there were also multiple w-box elements on _*pro*_*MaWRKYIII8* (Fig. S18). It is worth mentioning that the w-box element is not necessarily required to exist in the downstream gene promoter sequence in the *WRKY* transcription factor-dependent w-box element regulation pathway. Many *WRKY* transcription factors can bind to their own w-box for self-regulation or cross-regulation. The results of parsley ChIP-qPCR showed that *PcWRKY1* depended on the w-box element to bind itself and that the *PcWRKY3* and *PcPR1-1* promoters formed a cross network to regulate downstream reactions [68]. Arabidopsis *AtWRKY33* can form a positive feedback loop by binding to its own promoter [69]. This finding suggests that *WRKY* transcription factors are widely self-regulated and cross-regulated in vivo.

**Fig. 6:**
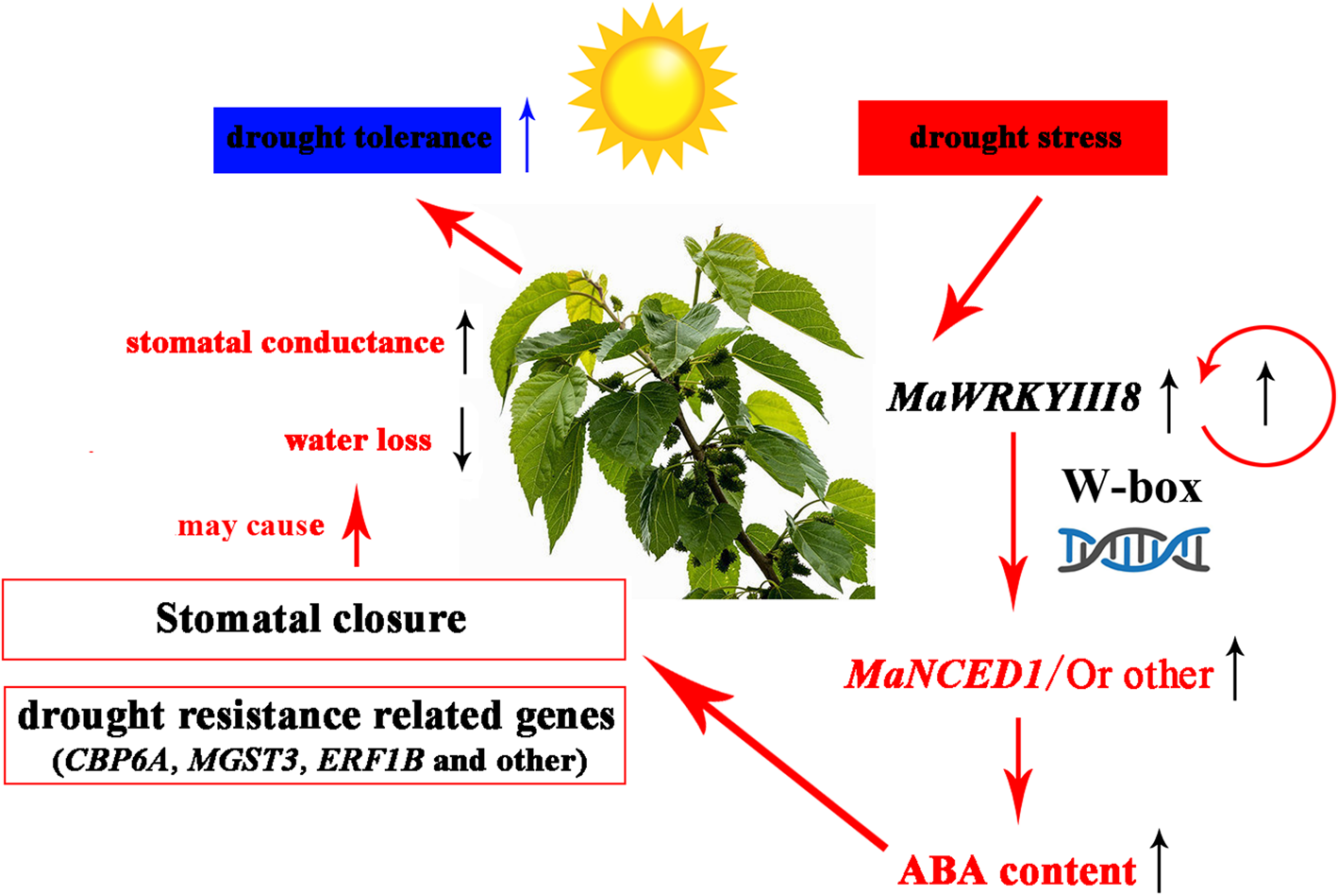
Molecular mechanism model of potential drought stress response involved in mulberry *MaWRKYIII8*

According to our research results, we drew a map of possible drought tolerance patterns of mulberry (Fig. 6). *MaWRKYIII8* expression was induced under drought stress, and *MaWRKYIII8* could regulate the expression of downstream ABA biosynthesis-related genes or bind with its own promoter for self-regulation (dependence on w-box). After ABA biosynthesis was activated in mulberry, some drought-responsive genes (such as *CBP6A*, *MGST3*, *ERF1B*, etc.) were activated. At the same time, stomatal opening and closing were also regulated, water loss was slowed down, and drought tolerance of mulberry was improved. However, the biological function of *MaWRKYIII8* involved in the mulberry drought stress response needs further verification.

## Conclusion

In this study, mulberry leaves under drought stress for 9 d were used as the experimental group, and normal-growing mulberry leaves were used as controls to perform correlation analysis of the transcriptome and proteome. We identified 9889 DEGs and 1893 DEPs, 818 of which could be correlated with each other. The expression pattern of the SGR gene in mulberry showed that *XM_ 010109758.1* was up-regulated in response to drought stress and involved in chloroplast degradation in response to premature senescence and yellowing of mulberry leaves under drought stress. The expression levels of *MaArg*, *MaP5CS1*, *MaproC*, *MaZEP*, *MaNCED* and other key enzyme genes were significantly upregulated, which activated the proline and ABA biosynthesis pathways and produced a large amount of proline and ABA to improve the drought tolerance of mulberry plants. In addition, *MaWRKYIII8* was up-regulated and induced by drought and exogenous ABA, indicating that *MaWRKYIII8* may be involved in the response process of mulberry to drought stress. Our work provides abundant gene resources for the study of drought tolerance in mulberry and lays a foundation for molecular genetic breeding in the future.

## Supplementary Information

**Additional file 1: Table S1.** Overview of proteome sequencing.

**Additional file 2: Table S2.** Statistics of NDEPs VS. DEGs comparison group members.

**Additional file 3: Table S3.** Statistics of DEPs VS. NDEGs comparison group members.

**Additional file 4: Table S4.** Top 20 KEGG pathways rich in co-differentially expressed genes.

**Additional file 5: Table S5.** Screening and identification of co-differentially expressed genes in ABA transduction pathway.

**Additional file 6: Table S6.** Screening and differential expression analysis of *WRKY* gene family in Mulberry.

**Additional file 7: Table S7.** Stay green rice genes related response of mulberry to drought stress.

**Additional file 8: Figure S1.** Correlation analysis among samples.

**Additional file 9: Figure S2.** FPKM distribution of unigenes in CK and DS9 samples of mulberry leaves.

**Additional file 10: Figure S3.** Cluster analysis of gene FPKM values among samples.

**Additional file 11: Figure S4.** Statistics of the number distribution of unigenes in different FPKM value ranges.

**Additional file 12: Figure S5.** Quality analysis of proteome sequencing. (a) Peptide length distribution map. (b) Protein molecular weight distribution. (c) Distribution of specific peptide number. (d) The protein coverage distribution was identified.

**Additional file 13: Figure S6.** Symmetric scatter plot of differentially expressed genes in CK vs. DS9 samples. The red triangle indicates the up-regulated gene, the blue square indicates the down-regulated gene, and the gray circle indicates that it is not a differentially expressed gene.

**Additional file 14: Figure S7.** Screening and identification of differentially expressed proteins by proteome sequencing.

**Additional file 15: Figure S8.** Prediction of subcellular localization of differentially expressed proteins.

**Additional file 16: Figure S9.** Enrichment analysis of differentially expressed genes in KOG database.

**Additional file 17: Figure S10.** GO enrichment analysis of differentially expressed proteins.

**Additional file 18: Figure S11.** Go enrichment analysis of a co-differentially expressed genes in transcriptome and proteome.

**Additional file 19: Figure S12.** KEGG enrichment analysis of co-differentially expressed gene.

**Additional file 20: Figure S13.** Expression patterns of ROS scavenging related genes in mulberry under drought stress. The abscissa indicated the days of drought stress treatment (0d, 3d, 6d, 9d, 12d), and the ordinate indicated the relative expression level of genes. DS0 is used as reference for each gene. *, *P* ≤ 0.05; **, *P* ≤ 0.01.

**Additional file 21: Figure S14.** Expression patterns of key enzyme genes of proline biosynthesis in mulberry under drought stress.

**Additional file 22: Figure S15.** Prediction of *MaWRKYIII8* interacting proteins by string database.

**Additional file 23: Figure S16.** Quantitative analysis of association between transcriptome and proteome members.

**Additional file 24: Figure S17.** Analysis of cis-acting elements in *MaNCED1* promoter sequence of mulberry.

**Additional file 25: Figure S18.** Analysis of cis-acting elements in *MaWRKYIII8* promoter sequence of mulberry.

## Data Availability

The data were deposited at NCBI’s Short Read Archive (SRA) database under the BioProject accession number PRJNA692033. The data will be released on 2022-02-14, before which information can be obtained through the link below (https://dataview.ncbi.nlm.nih.gov/object/PRJNA692033?reviewer=ep63ethcm92vv71 elr8n20t725).
