# Supplementary material for: Molecular mechanism of mulberry response to drought stress revealed by complementary transcriptomic and iTRAQ analyses
Source: BMC Plant Biol. 2022 Jan 17;22:36. doi: 10.1186/s12870-021-03410-x (PMC8762937; doi:10.1186/s12870-021-03410-x)
Supplement: Supplementary file 1 — Additional file 1: Table S1. Overview of proteome sequencing. [file 12870_2021_3410_MOESM1_ESM.docx]

| Total spectra | Know Spectra | Unique Spetra | Peptide | Unique Peptide | Protein |
| --- | --- | --- | --- | --- | --- |
| 878,525 | 55,412 | 50,481 | 24,926 | 23,442 | 6,188 |

Table S1. Overview of proteome sequencing.
