# Supplementary material for: Molecular mechanism of mulberry response to drought stress revealed by complementary transcriptomic and iTRAQ analyses
Source: BMC Plant Biol. 2022 Jan 17;22:36. doi: 10.1186/s12870-021-03410-x (PMC8762937; doi:10.1186/s12870-021-03410-x)
Supplement: Supplementary file 4 — Additional file 4: Table S4. Top 20 KEGG pathways rich in co-differentially expressed genes. [file 12870_2021_3410_MOESM4_ESM.docx]

| Serial number | Pathway ID | Pathway Term | Number of Correlation |
| --- | --- | --- | --- |
| 1 | ko01100 | Metabolic pathways | 234 |
| 2 | ko01110 | Biosynthesis of secondary metabolites | 140 |
| 3 | ko01230 | Biosynthesis of amino acids | 39 |
| 4 | ko01200 | Carbon metabolism | 34 |
| 5 | ko00500 | Starch and sucrose metabolism | 27 |
| 6 | ko00520 | Amino sugar and nucleotide sugar metabolism | 25 |
| 7 | ko04141 | Protein processing in endoplasmic reticulum | 24 |
| 8 | ko00940 | Phenylpropanoid biosynthesis | 24 |
| 9 | ko00010 | Glycolysis / Gluconeogenesis | 22 |
| 10 | ko04626 | Plant-pathogen interaction | 21 |
| 11 | ko00561 | Glycerolipid metabolism | 19 |
| 12 | ko03010 | Ribosome | 16 |
| 13 | ko03008 | Ribosome biogenesis in eukaryotes | 15 |
| 14 | ko00230 | Purine metabolism | 14 |
| 15 | ko00270 | Cysteine and methionine metabolism | 14 |
| 16 | ko00052 | Galactose metabolism | 14 |
| 17 | ko03018 | RNA degradation | 14 |
| 18 | ko00620 | Pyruvate metabolism | 14 |
| 19 | ko04146 | Peroxisome | 14 |
| 20 | ko04144 | Endocytosis | 12 |
