# Supplementary material for: Molecular mechanism of mulberry response to drought stress revealed by complementary transcriptomic and iTRAQ analyses
Source: BMC Plant Biol. 2022 Jan 17;22:36. doi: 10.1186/s12870-021-03410-x (PMC8762937; doi:10.1186/s12870-021-03410-x)
Supplement: Supplementary file 5 — Additional file 5: Table S5. Screening and identification of co-differentially expressed genes in ABA transduction pathway. [file 12870_2021_3410_MOESM5_ESM.docx]

| Gene name | Genome ID | Drought stress response related genes | | |
| --- | --- | --- | --- | --- |
|  |  | CK | DS9 | UP or DOWN |
| *MaPYL1* | XM_010111579.1 | 0 | 5.76 | UP |
| *MaPYL2* | XM_010110850.1 | 4.61 | 0.34 | DOWN |
| *MaPYL3* | XM_010089239.1 | 0.52 | 0.07 | DOWN |
| *MaPP2C* | XM_010097045.1 | 3.14 | 24.81 | UP |
| *MaABF1* | XM_010113887.1 | 0 | 1.18 | UP |
| *MaABF2* | XM_010108602.1 | 0.54 | 0 | DOWN |
| *MaABF3* | XM_010111823.1 | 0.68 | 5.11 | UP |
