# Supplementary material for: Molecular mechanism of mulberry response to drought stress revealed by complementary transcriptomic and iTRAQ analyses
Source: BMC Plant Biol. 2022 Jan 17;22:36. doi: 10.1186/s12870-021-03410-x (PMC8762937; doi:10.1186/s12870-021-03410-x)
Supplement: Supplementary file 6 — Additional file 6: Table S6. Screening and differential expression analysis of WRKY gene family in Mulberry. [file 12870_2021_3410_MOESM6_ESM.docx]

| Gene family naming | Number | DNGs  UP or DOWN | DNPS  UP or DOWN |
| --- | --- | --- | --- |
| *MaWRKYI1* | XP_010112795.1 | * | * |
| *MaWRKYI2* | XP_010111075.1 | * | * |
| *MaWRKYI3* | XP_010108734.1 | UP | UP |
| *MaWRKYI4* | XP_010100844.1 | * | * |
| *MaWRKYI5* | XP_010089564.1 | UP | UP |
| *MaWRKYI6* | XP_010092241.1 | * | * |
| *MaWRKYI7* | XP_010093567.1 | UP | * |
| *MaWRKYI8* | XP_010097277.1 | * | * |
| *MaWRKYI9* | XP_010099437.1 | UP | * |
| *MaWRKYIIa1* | XP_010092197.1 | UP | * |
| *MaWRKYIIa2* | XP_010101038.1 | UP | * |
| *MaWRKYIIa3* | XP_010101037.1 | UP | * |
| *MaWRKYIIb1* | XP_010099182.1 | UP | * |
| *MaWRKYIIb2* | XP_010096349.1 | * | * |
| *MaWRKYIIb3* | XP_010096871.1 | UP | * |
| *MaWRKYIIb4* | XP_010089474.1 | UP | * |
| *MaWRKYIIb5* | XP_010104966.1 | * | * |
| *MaWRKYIIb6* | XP_010102869.1 | * | * |
| *MaWRKYIIb7* | XP_010103852.1 | Down | * |
| *MaWRKYIIc1* | XP_010086980.1 | * | * |
| *MaWRKYIIc2* | XP_010090653.1 | UP | * |
| *MaWRKYIIc3* | XP_010091969.1 | * | * |
| *MaWRKYIIc4* | XP_010086778.1 | * | * |
| *MaWRKYIIc5* | XP_010112967.1 | * | * |
| *MaWRKYIIc6* | XP_010112390.1 | UP | * |
| *MaWRKYIIc7* | XP_010109362.1 | * | * |
| *MaWRKYIIc8* | XP_010108555.1 | * | * |
| *MaWRKYIIc9* | XP_010107303.1 | * | * |
| *MaWRKYIIc10* | XP_010106707.1 | UP | * |
| *MaWRKYIIc11* | XP_010102144.1 | * | * |
| *MaWRKYIIc12* | XP_010086912.1 | * | * |
| *MaWRKYIIc13* | XP_010086980.1 | * | * |
| *MaWRKYIIc14* | XP_010100977.1 | UP |  |
| *MaWRKYIId1* | XP_010100083.1 | * | * |
| *MaWRKYIId2* | XP_010093521.1 | * | * |
| *MaWRKYIId3* | XP_010093526.1 | * | * |
| *MaWRKYIId4* | XP_010112111.1 | * | * |
| *MaWRKYIId5* | XP_010093696.1 | Down | * |
| *MaWRKYIIe1* | XP_010098516.1 | * | * |
| *MaWRKYIIe2* | XP_010087230.1 | * | * |
| *MaWRKYIIe3* | XP_010092573.1 | * | * |
| *MaWRKYIIe4* | XP_010112624.1 | * | * |
| *MaWRKYIIe5* | XP_010105730.1 | * | * |
| *MaWRKYIIe6* | XP_010102121.1 | * | * |
| *MaWRKYIIe7* | XP_010086863.1 | * | * |
| *MaWRKYIII1* | XP_010097859.1 | * | * |
| *MaWRKYIII2* | XP_010098011.1 | Down | * |
| *MaWRKYIII3* | XP_010093540.1 | * | * |
| *MaWRKYIII4* | XP_010095789.1 | * | * |
| *MaWRKYIII5* | XP_010093537.1 | Down | * |
| *MaWRKYIII6* | XP_010103666.1 | * | * |
| *MaWRKYIII7* | XP_010103665.1 | Up | * |
| *MaWRKYIII8* | XP_010104968.1 | Up | Up |
