# Supplementary material for: Molecular mechanism of mulberry response to drought stress revealed by complementary transcriptomic and iTRAQ analyses
Source: BMC Plant Biol. 2022 Jan 17;22:36. doi: 10.1186/s12870-021-03410-x (PMC8762937; doi:10.1186/s12870-021-03410-x)
Supplement: Supplementary file 7 — Additional file 7: Table S7. Stay green rice genes related response of mulberry to drought stress. [file 12870_2021_3410_MOESM7_ESM.docx]

| *AtSGR* | Stay green rice genes in mulberry | | | | DGEs |
| --- | --- | --- | --- | --- | --- |
|  | *MaSGR* | Genome ID | CK FPKM | DS9 FPKM |  |
| *AtSGR1* | *MaSGR1* | XM_010101265.1  XM_010109758.1  XM_010107031.1 | 11.887 | 5.237 | down |
| *AtSGR2* | *MaSGR2* |  | 5.043 | 48.630 | up |
| *AtSGR-like* | *MaSGR-like* |  | 0.470 | 0.270 | down |
