# Supplementary figures and images for: Molecular mechanism of mulberry response to drought stress revealed by complementary transcriptomic and iTRAQ analyses
Source: BMC Plant Biol. 2022 Jan 17;22:36. doi: 10.1186/s12870-021-03410-x (PMC8762937; doi:10.1186/s12870-021-03410-x)

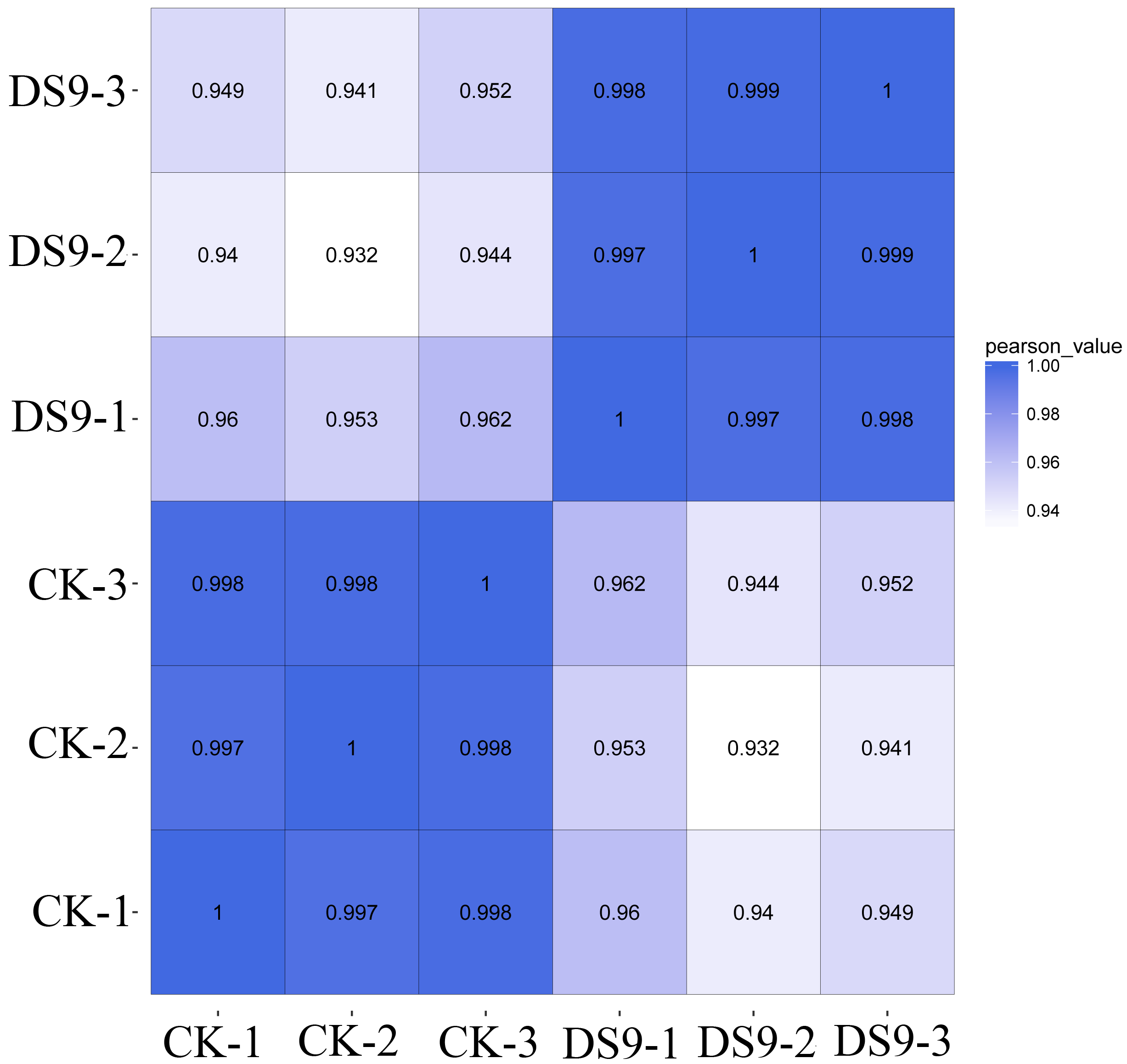

Supplement: Supplementary file 8 — Additional file 8: Figure S1. Correlation analysis among samples. [file 12870_2021_3410_MOESM8_ESM.tiff]

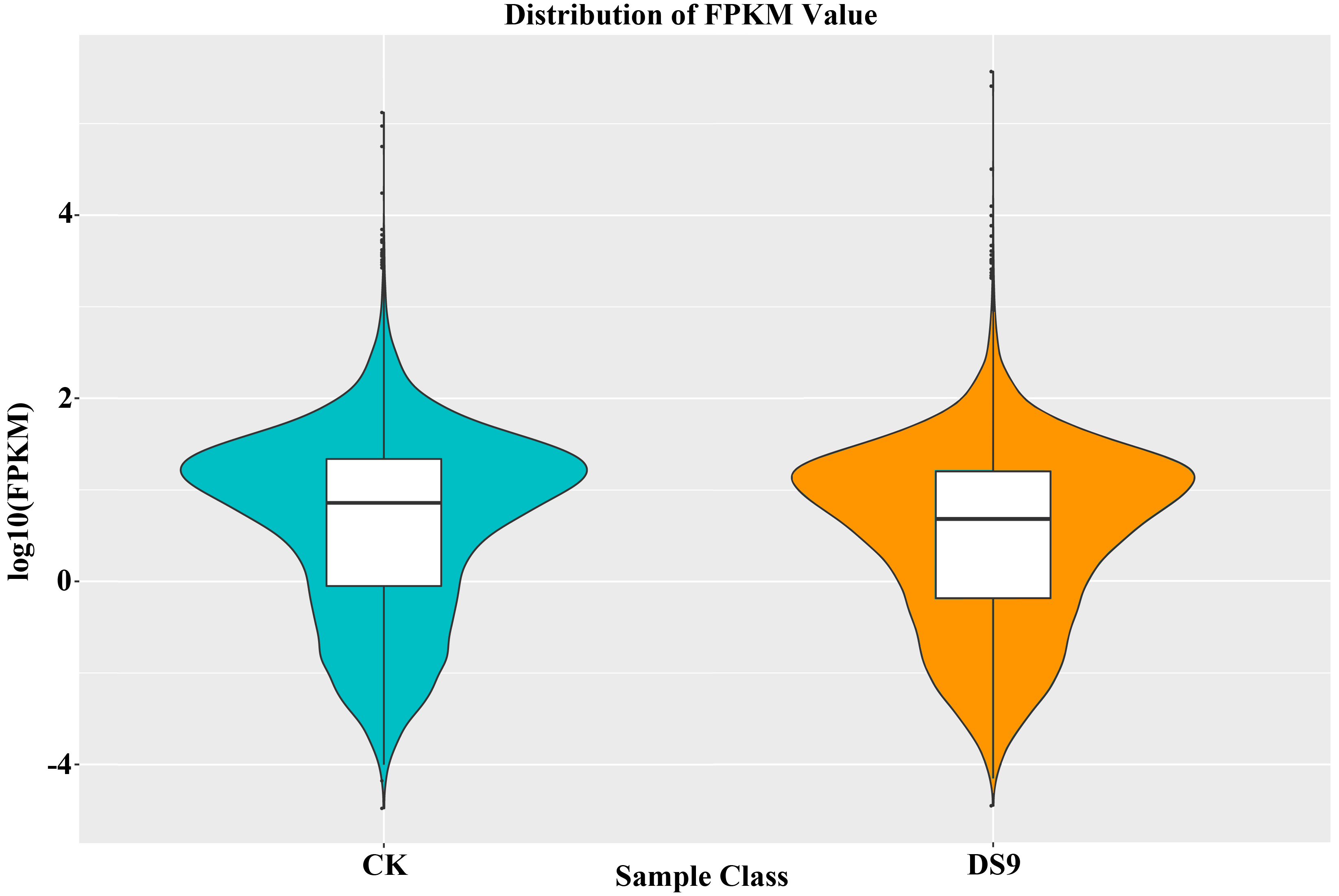

Supplement: Supplementary file 9 — Additional file 9: Figure S2. FPKM distribution of unigenes in CK and DS9 samples of mulberry leaves. [file 12870_2021_3410_MOESM9_ESM.tiff]

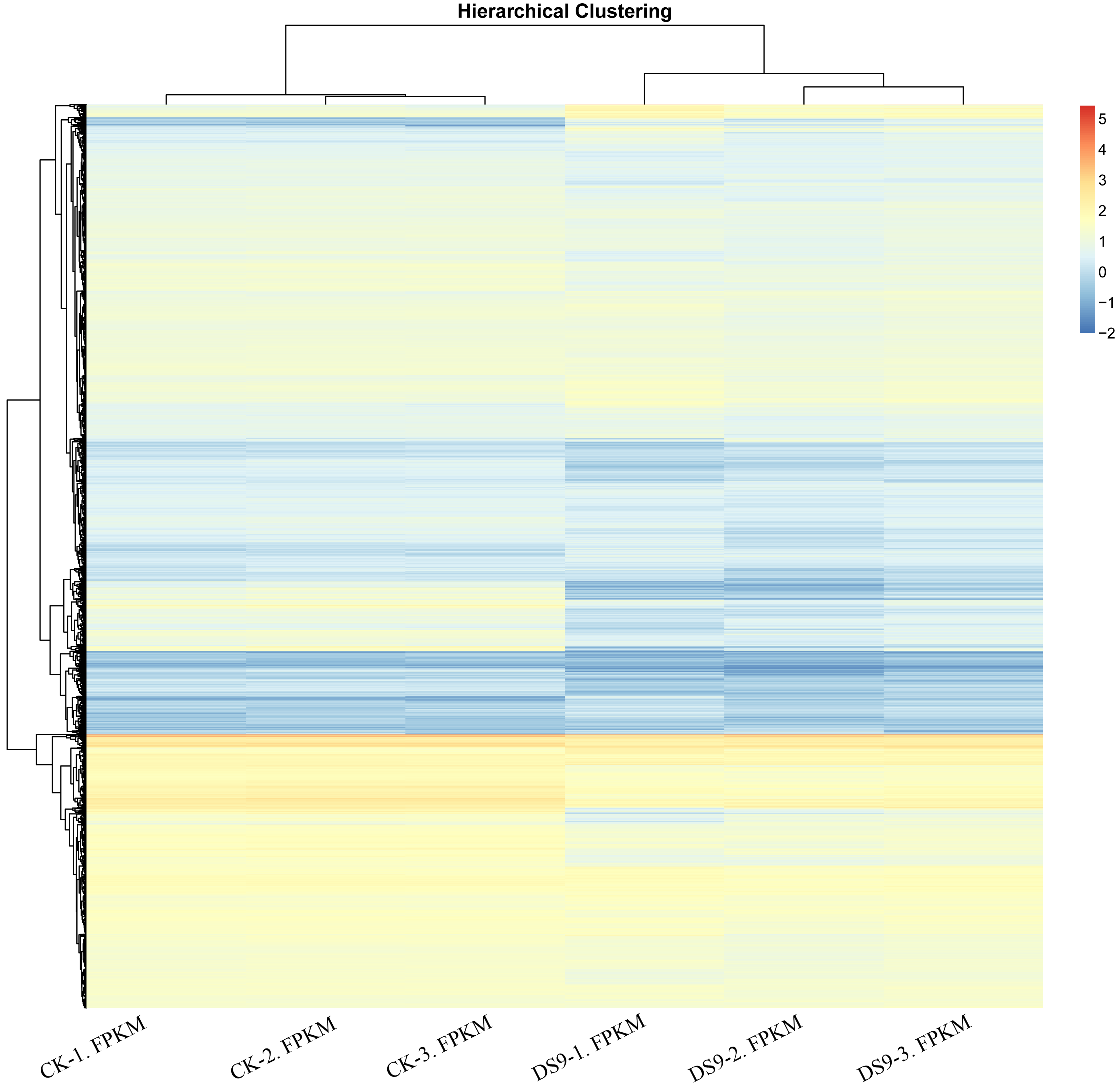

Supplement: Supplementary file 10 — Additional file 10: Figure S3. Cluster analysis of gene FPKM values among samples. [file 12870_2021_3410_MOESM10_ESM.tif]

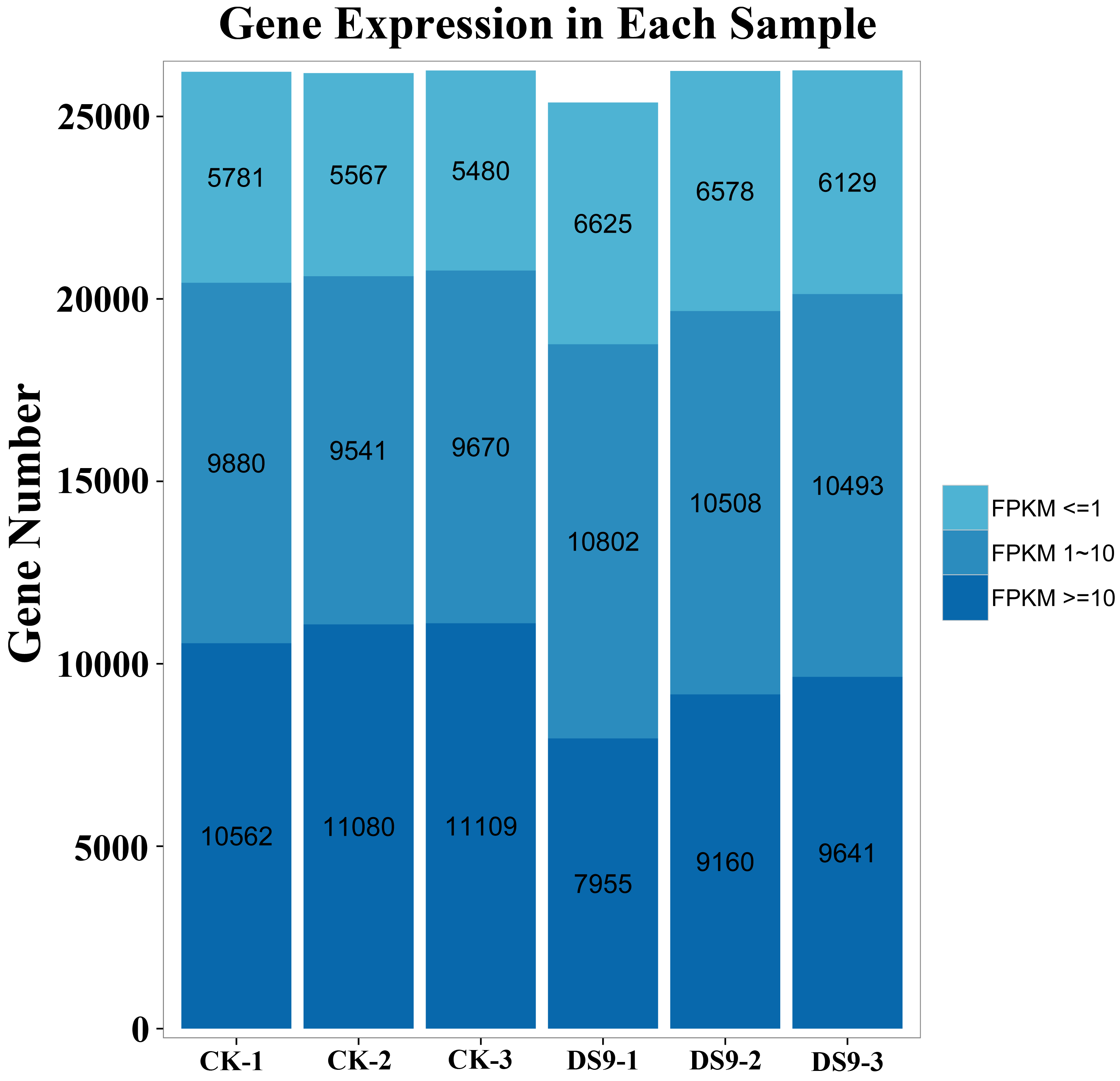

Supplement: Supplementary file 11 — Additional file 11: Figure S4. Statistics of the number distribution of unigenes in different FPKM value ranges. [file 12870_2021_3410_MOESM11_ESM.tiff]

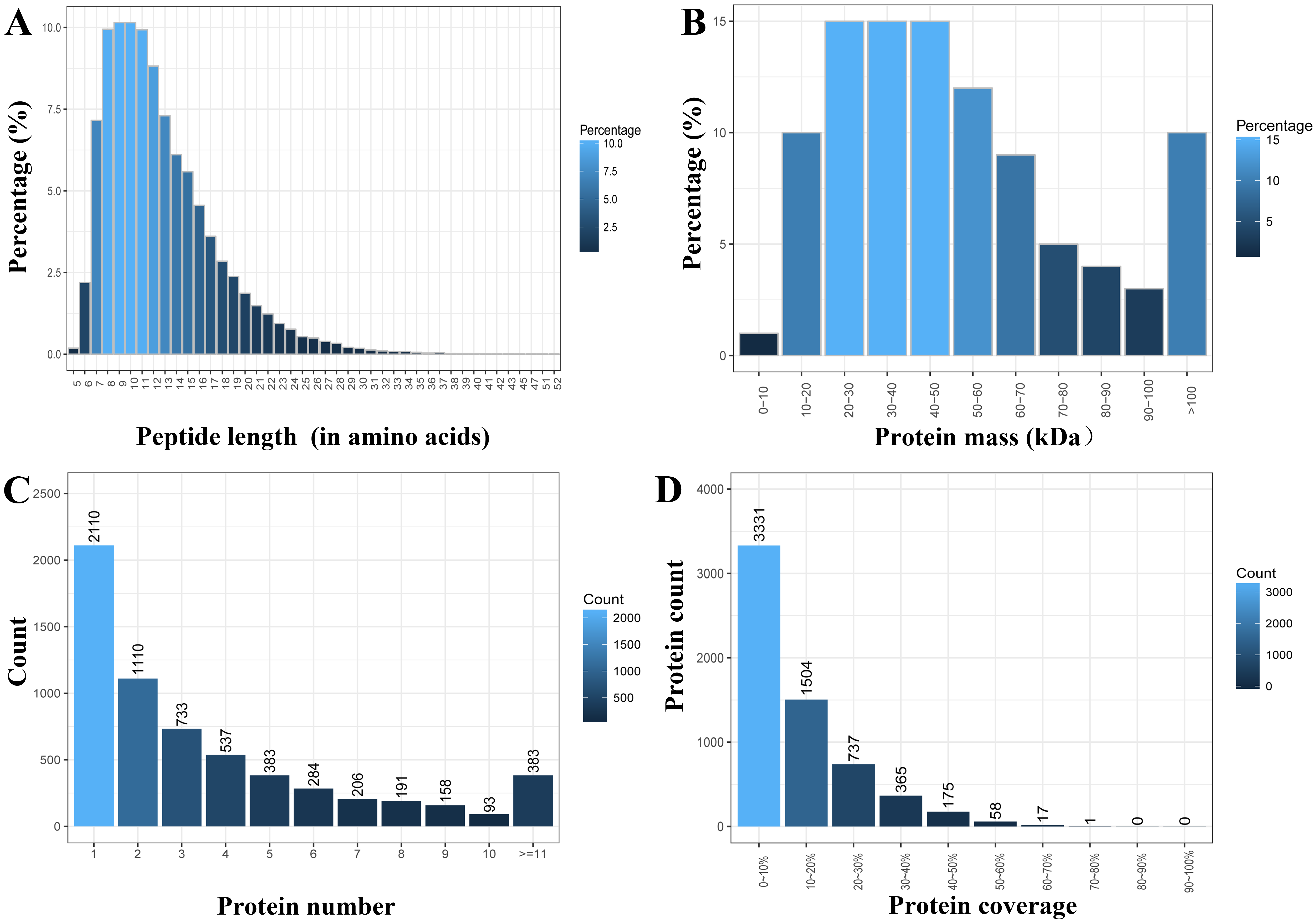

Supplement: Supplementary file 12 — Additional file 12: Figure S5. Quality analysis of proteome sequencing. (a) Peptide length distribution map. (b) Protein molecular weight distribution. (c) Distribution of specific peptide number. (d) The protein coverage distribution was identified. [file 12870_2021_3410_MOESM12_ESM.tiff]

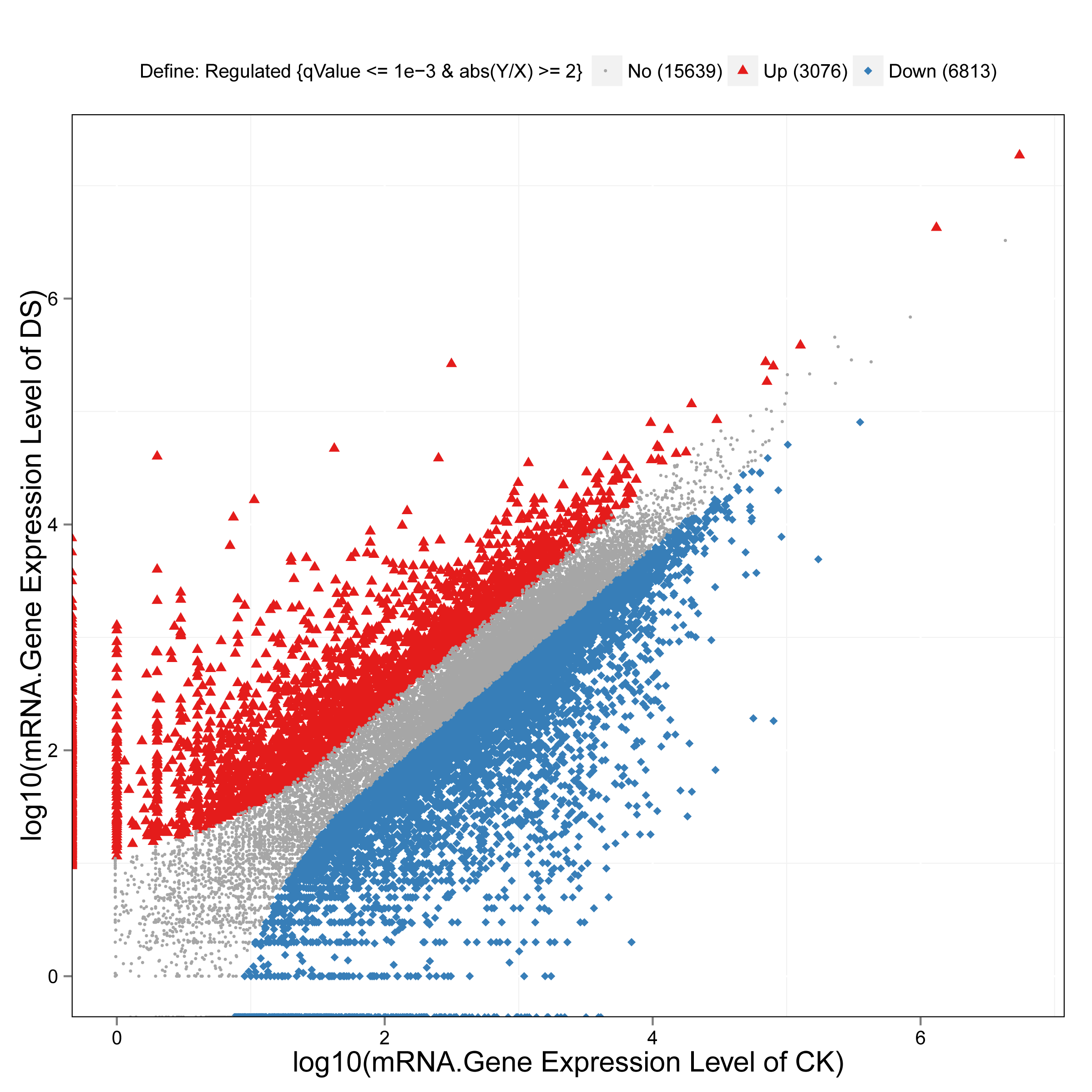

Supplement: Supplementary file 13 — Additional file 13: Figure S6. Symmetric scatter plot of differentially expressed genes in CK vs. DS9 samples. The red triangle indicates the up-regulated gene, the blue square indicates the down-regulated gene, and the gray circle indicates that it is not a differentially expressed gene. [file 12870_2021_3410_MOESM13_ESM.tif]

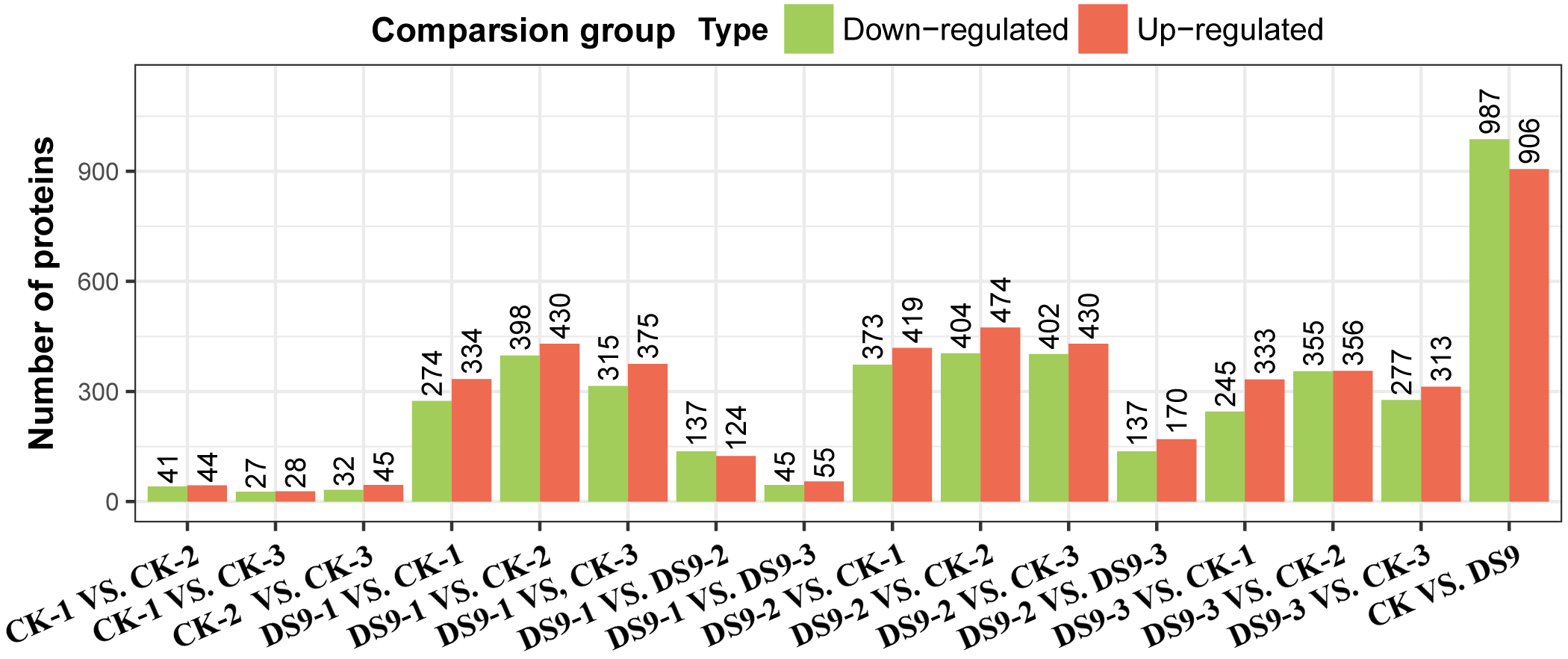

Supplement: Supplementary file 14 — Additional file 14: Figure S7. Screening and identification of differentially expressed proteins by proteome sequencing. [file 12870_2021_3410_MOESM14_ESM.tif]

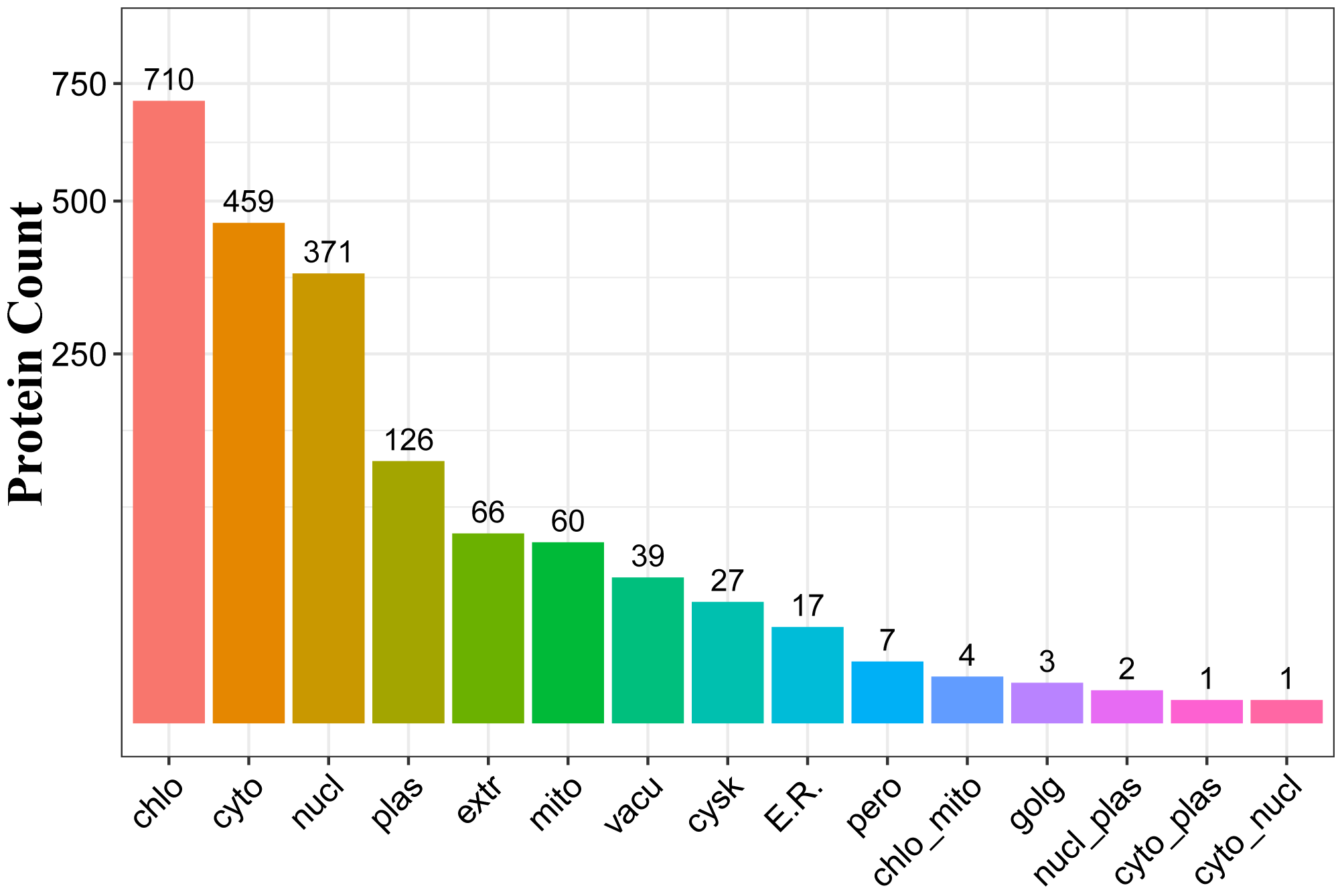

Supplement: Supplementary file 15 — Additional file 15: Figure S8. Prediction of subcellular localization of differentially expressed proteins. [file 12870_2021_3410_MOESM15_ESM.tif]

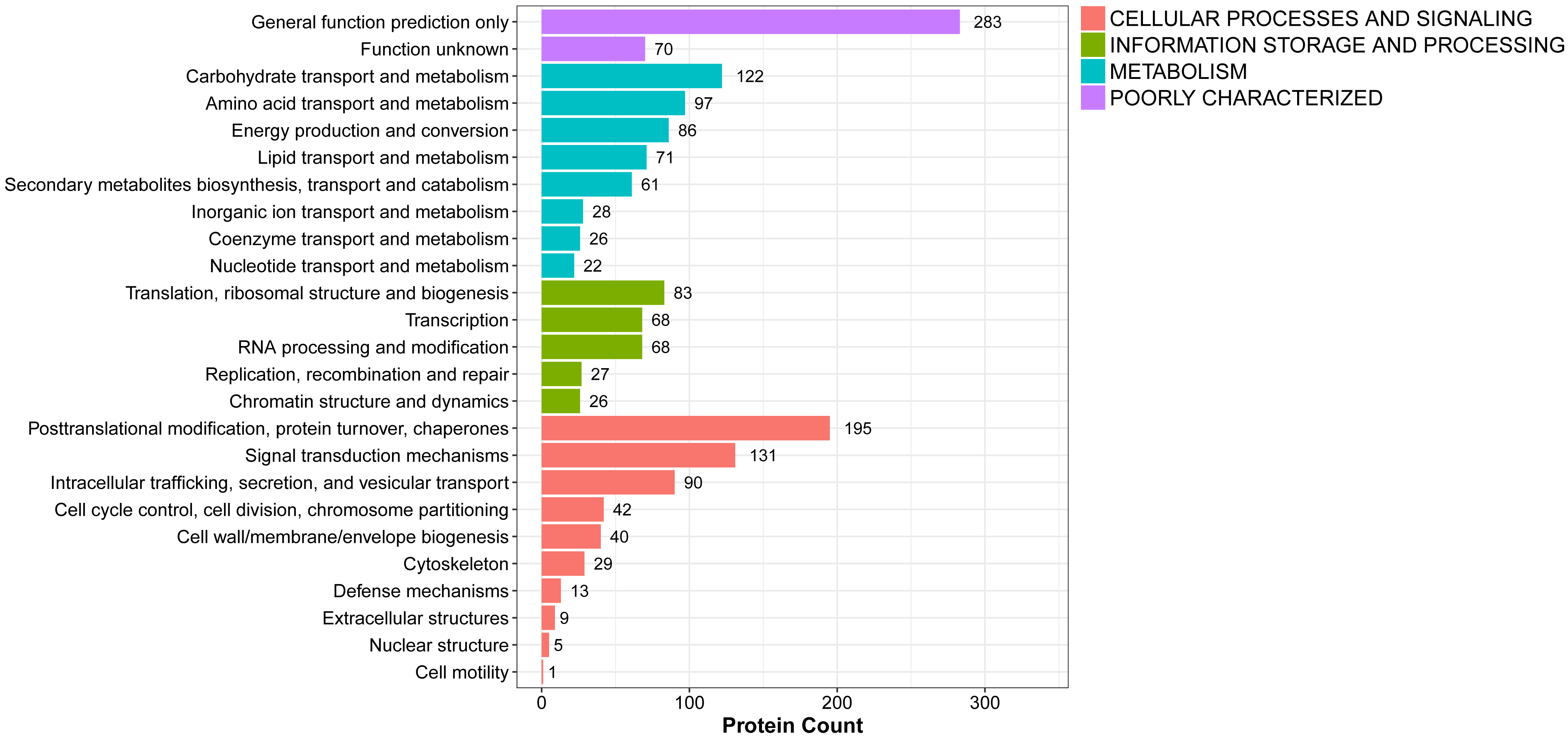

Supplement: Supplementary file 16 — Additional file 16: Figure S9. Enrichment analysis of differentially expressed genes in KOG database. [file 12870_2021_3410_MOESM16_ESM.tif]

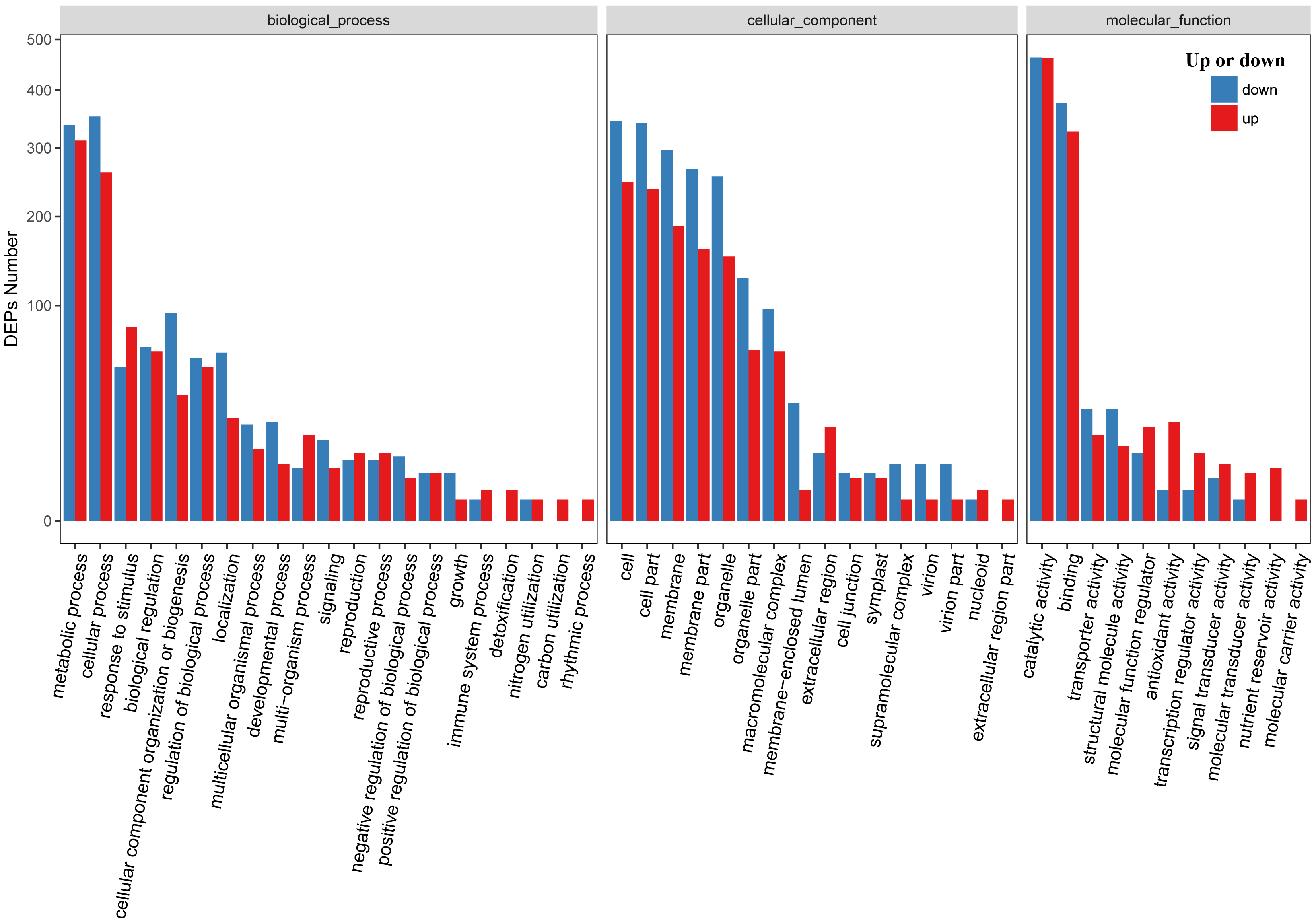

Supplement: Supplementary file 17 — Additional file 17: Figure S10. GO enrichment analysis of differentially expressed proteins. [file 12870_2021_3410_MOESM17_ESM.tif]

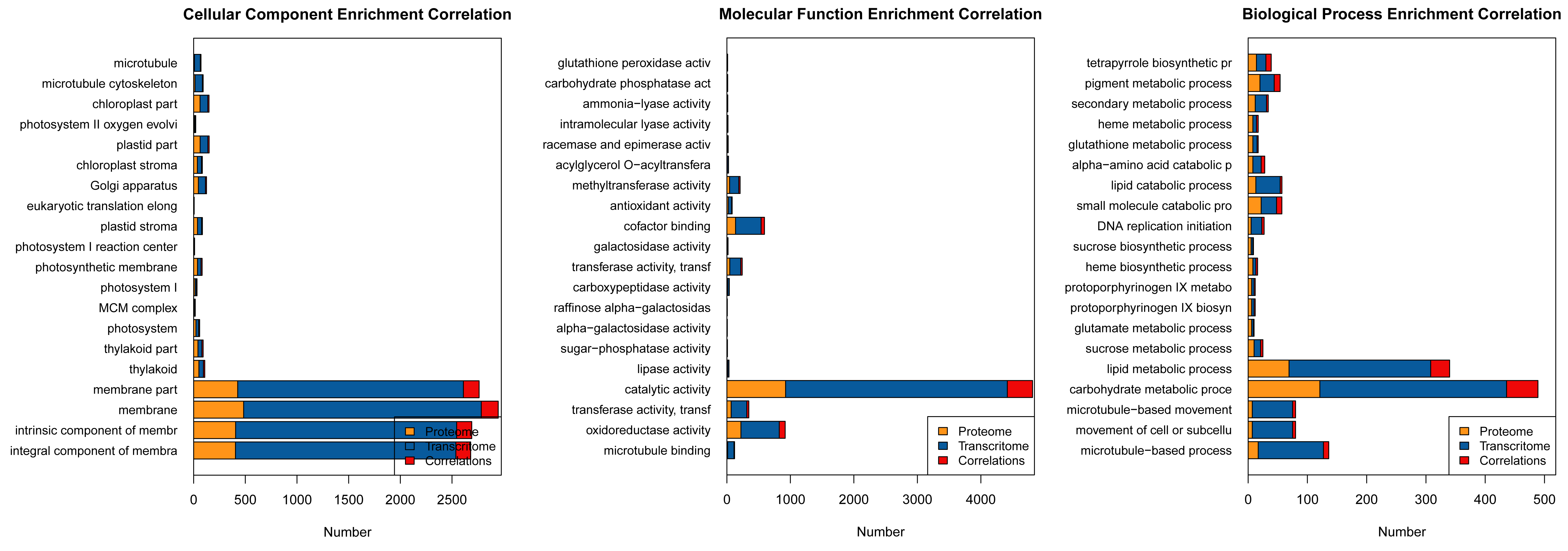

Supplement: Supplementary file 18 — Additional file 18: Figure S11. Go enrichment analysis of a co-differentially expressed genes in transcriptome and proteome. [file 12870_2021_3410_MOESM18_ESM.tif]

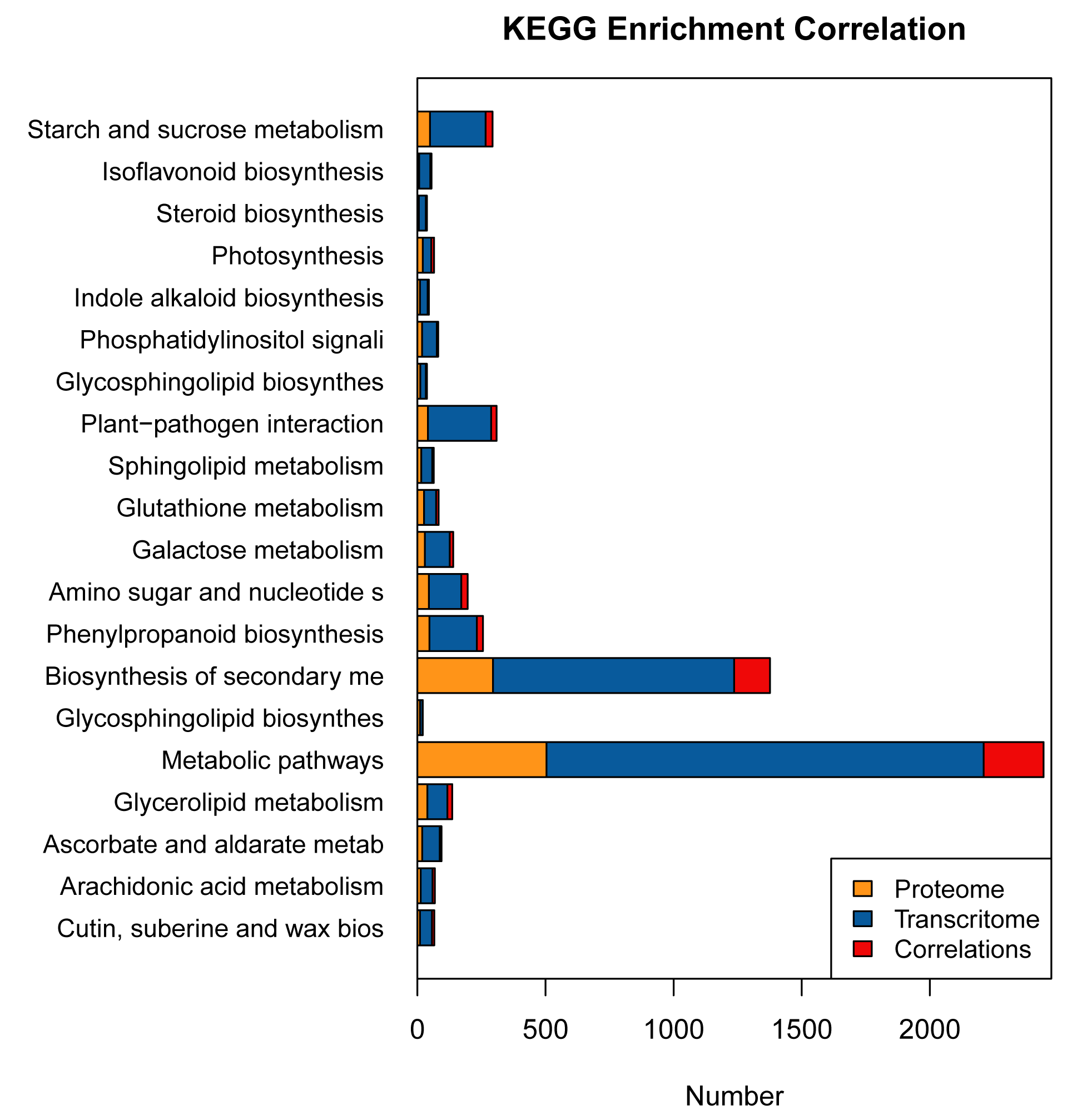

Supplement: Supplementary file 19 — Additional file 19: Figure S12. KEGG enrichment analysis of co-differentially expressed gene. [file 12870_2021_3410_MOESM19_ESM.tif]

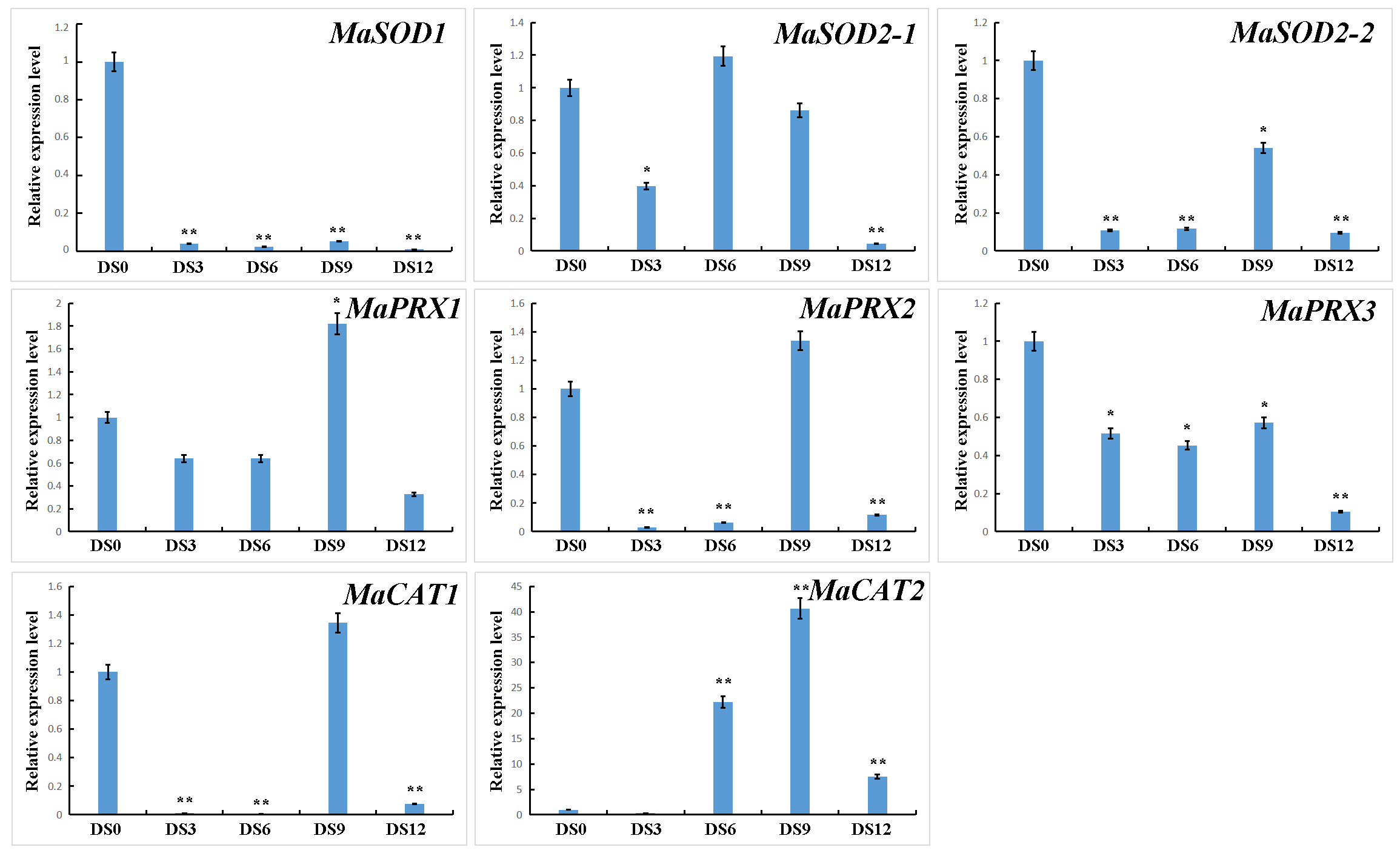

Supplement: Supplementary file 20 — Additional file 20: Figure S13. Expression patterns of ROS scavenging related genes in mulberry under drought stress. The abscissa indicated the days of drought stress treatment (0d, 3d, 6d, 9d, 12d), and the ordinate indicated the relative expression level of genes. DS0 is used as reference for each gene. *, P ≤ 0.05; **, P ≤ 0.01. [file 12870_2021_3410_MOESM20_ESM.tif]

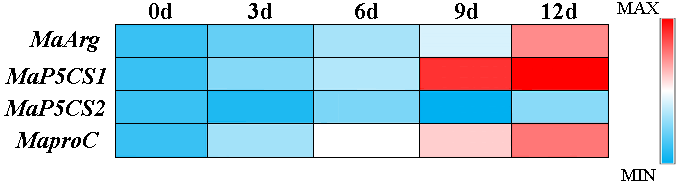

Supplement: Supplementary file 21 — Additional file 21: Figure S14. Expression patterns of key enzyme genes of proline biosynthesis in mulberry under drought stress. [file 12870_2021_3410_MOESM21_ESM.tif]

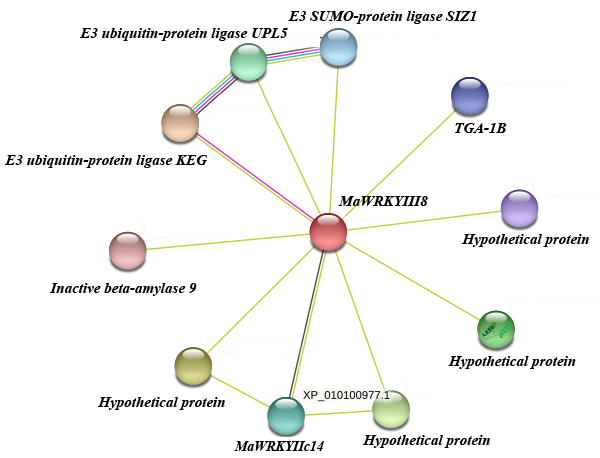

Supplement: Supplementary file 22 — Additional file 22: Figure S15. Prediction of MaWRKYIII8 interacting proteins by string database. [file 12870_2021_3410_MOESM22_ESM.tif]

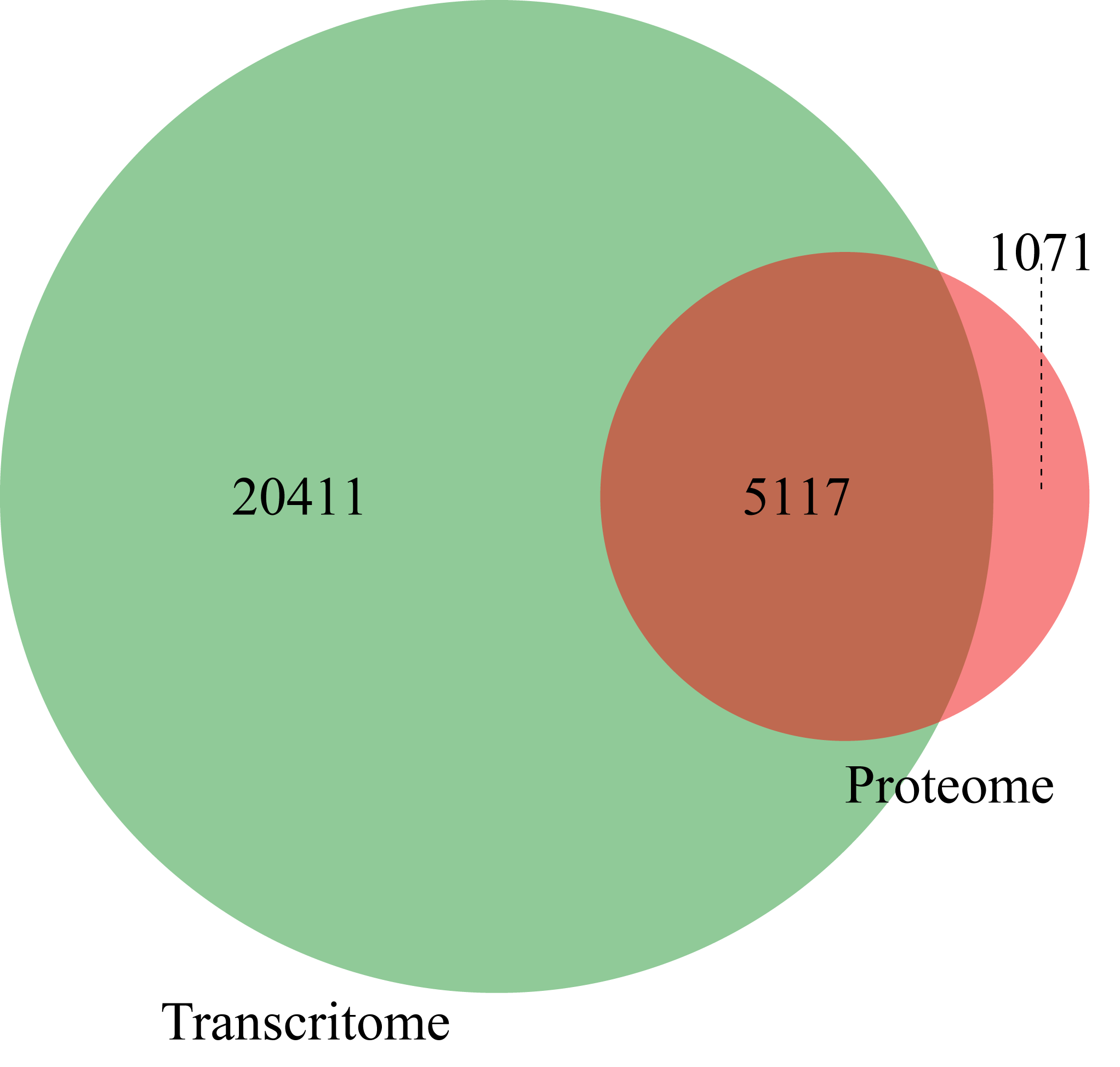

Supplement: Supplementary file 23 — Additional file 23: Figure S16. Quantitative analysis of association between transcriptome and proteome members. [file 12870_2021_3410_MOESM23_ESM.tif]

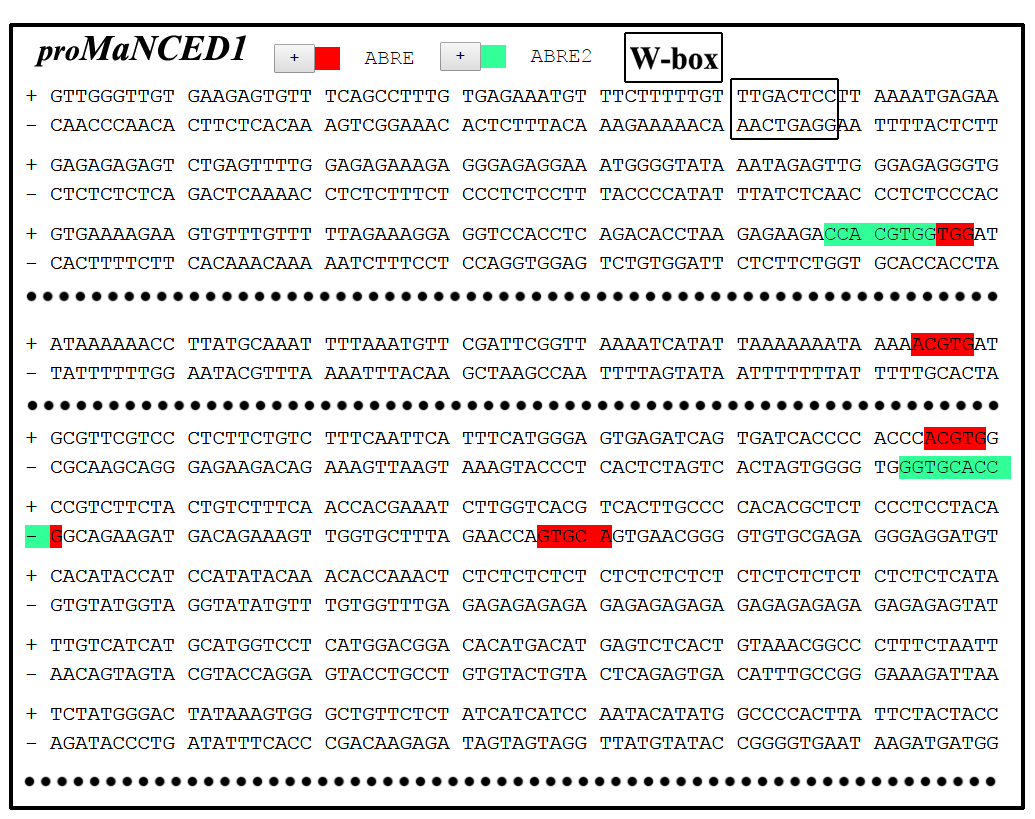

Supplement: Supplementary file 24 — Additional file 24: Figure S17. Analysis of cis-acting elements in MaNCED1 promoter sequence of mulberry. [file 12870_2021_3410_MOESM24_ESM.tif]

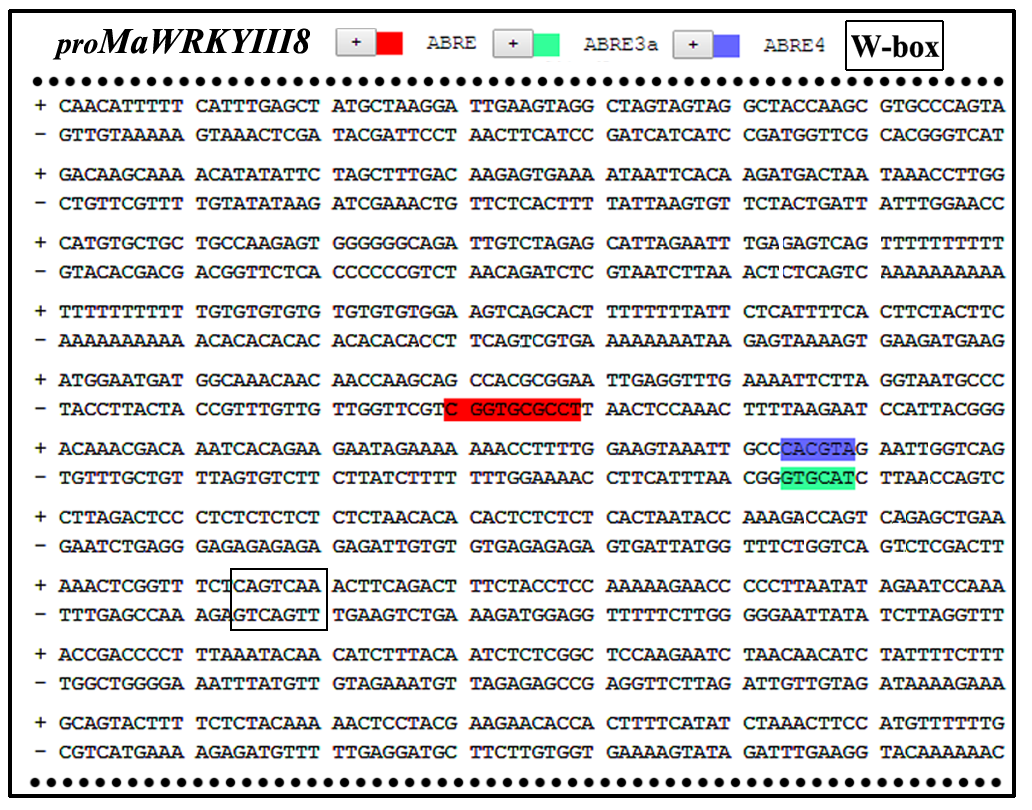

Supplement: Supplementary file 25 — Additional file 25: Figure S18. Analysis of cis-acting elements in MaWRKYIII8 promoter sequence of mulberry. [file 12870_2021_3410_MOESM25_ESM.tiff]
